# Impact of the Coformer Carbon-Chain Length on the Properties of Haloperidol Pharmaceutical Salts

**DOI:** 10.1021/acs.cgd.5c00251

**Published:** 2025-04-28

**Authors:** Francisco J. Acebedo-Martínez, Carolina Alarcón-Payer, Alicia Domínguez-Martín, Antonio Frontera, Cristóbal Verdugo-Escamilla, Duane Choquesillo-Lazarte

**Affiliations:** † Laboratorio de Estudios Cristalográficos, IACT-CSIC, Avda. de las Palmeras 4, 18100 Armilla, Spain; ‡ Servicio de Farmacia, Hospital Universitario Virgen de las Nieves, 18014 Granada, Spain; § Department of Inorganic Chemistry, Faculty of Pharmacy, University of Granada, 18071 Granada, Spain; ∥ Departament de Química, Universitat de les Illes Balears, Crta. de Valldemossa km 7.5, 07122 Palma, Spain

## Abstract

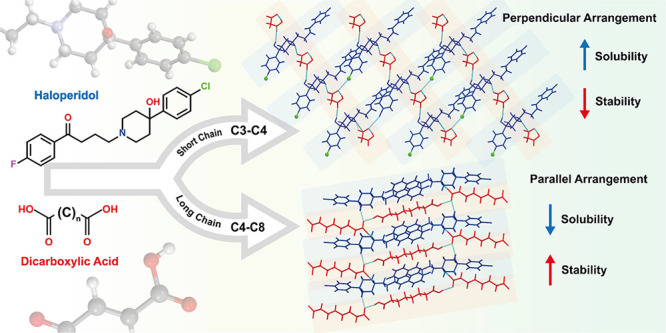

Haloperidol (HAL) is a conventional antipsychotic drug with poor aqueous solubility, which is associated with a major risk of side effects. In this context, crystal engineering has provided an efficient approach for tuning the physicochemical properties of active pharmaceutical ingredients (APIs). However, there is a huge lack of knowledge about how coformer molecules impact the pharmaceutical properties of the multicomponent materials, with special attention to solubility and stability. To this purpose, five novel salts and three ionic cocrystals were synthesized using HAL and a series of closely related dicarboxylic acid counterions. Mechanochemical strategies were applied for synthesis, while thermal, spectroscopic, and X-ray diffraction techniques were used for a complete characterization of the materials. By understanding the relationships between the crystal structures and the final properties, this research seeks to inform the rational design of HAL multicomponent drugs, providing a framework for improving the performance of not only HAL but also other APIs with similar challenges.

## Abstract 2

This study explores the impact of carbon-chain length in dicarboxylic acid coformers on the structure and properties of haloperidol salts and ionic cocrystals. Evidence from X-ray diffraction, DFT calculations, and thermal analysis supports a rational coformer selection strategy to enhance solubility and stability in haloperidol multicomponent materials.

## 1 Introduction

Since its introduction in the late 1950s, Haloperidol (HAL) has been a well-established antipsychotic drug for the treatment of schizophrenia, acute psychosis, and severe behavioral disturbances.^1−3^ Despite its clinical significance, demonstrated by its inclusion in the World Health Organization’s List of Essential Medicines, the poor aqueous solubility of the drug significantly limits its therapeutic potential.^2,4^

As a Class II drug under the Biopharmaceutics Classification System (BCS), HAL exhibits high permeability but poor solubility in aqueous solution, leading to suboptimal dissolution in the gastrointestinal tract and reduced oral bioavailability.^5,6^ These limitations often necessitate higher doses to achieve therapeutic plasma concentrations, increasing the risk of dose-dependent adverse effects, including extrapyramidal symptoms, drowsiness, and tardive dyskinesia.^7−10^ Addressing these solubility-related challenges is critical for improving HAL’s performance, patient compliance, and therapeutic outcomes.

During the last decades, the development of pharmaceutical multicomponent materials (PMMs) has offered a promising strategy for enhancing the solubility and other physicochemical properties of different active pharmaceutical ingredients (APIs).^11−13^ PMMs are described as crystalline materials consisting of, at least, one API and a coformer molecule in a stoichiometric ratio. Coformers are incorporated into the crystal lattice through noncovalent interactions, such as hydrogen bonds, creating a new crystal structure with unique physicochemical properties.^14−16^

Nowadays, the development of PMMs is a fundamental research area in the pharmaceutical industry.^17^ However, a critical, yet poorly explored, factor in the rational design of salts and cocrystals is the impact of coformers’ carbon chain length on the final properties of the material.^18^ The carbon chain length influences the spatial arrangement of molecules, the strength and nature of intermolecular interactions, and the hydrophobicity of the resulting structure, thereby having a huge impact on the final physicochemical properties of PMMs.^19−21^

**Figure 1:**
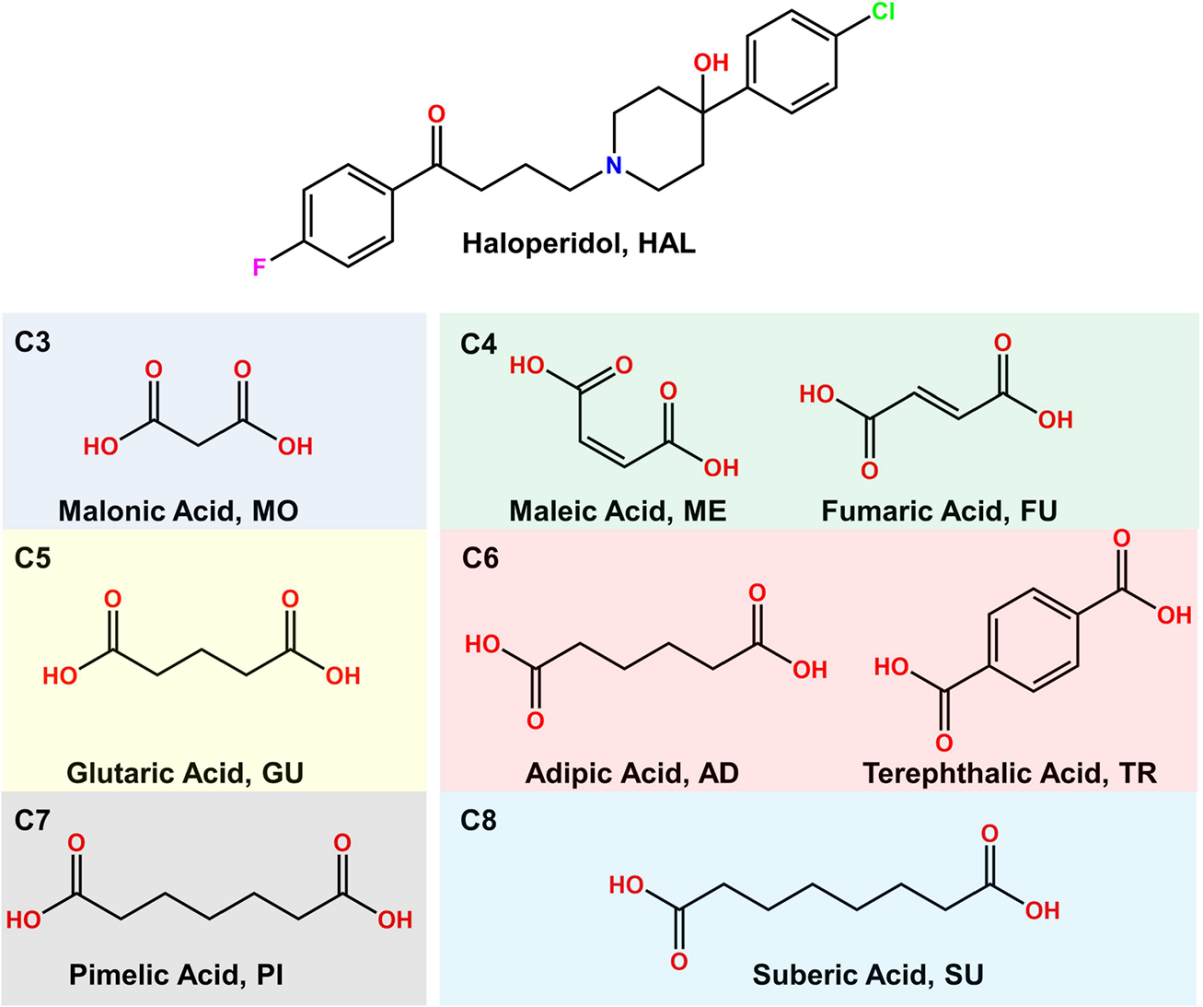
Chemical formula of haloperidol (HAL), malonic acid (MO), maleic acid (ME), fumaric acid (FU), glutaric acid (GU), adipic acid (AD), terephthalic acid (TR), pimelic acid (PI) and suberic acid (SU).

This study aims to investigate the influence of coformers’ carbon chain length on the properties of a series of HAL salts and ionic cocrystals. To this end, a range of structurally diverse coformers, including short-chain diacids (malonic, maleic, fumaric), medium-chain diacids (glutaric, adipic, pimelic, suberic), and an aromatic diacid (terephthalic) are employed (Figure 1). Short-chain diacids are expected to form compact, stable lattices due to their size and acidity, while medium-chain diacids offer varied chain lengths that influence packing efficiency and flexibility.^20,22^ Aromatic diacids introduce π–π stacking and robust hydrogen bonding, potentially enhancing thermal stability and mechanical strength.^23−26^

Advanced characterization techniques will be used to comprehensively assess the effects of carbon chain length. Molecular recognition has been thoroughly characterized by single-crystal X-ray diffraction and computational methods. In addition, the influence of such structural changes on different physicochemical properties has been also evaluated through different analytical techniques.

By understanding these relationships, this research seeks to inform the rational coformer selection for designing HAL pharmaceutical salts, providing a framework for improving the performance of not only HAL but also other APIs with similar challenges.

## 2 Experimental Section

### 2.1 Synthesis of Cocrystals

#### 2.1.1 Mechanochemical Synthesis

The mechanochemical synthesis of the new solids was carried out via Liquid-Assisted Grinding (LAG) in a Retsch MM2000 ball mill operating at a frequency of 25 Hz, during 30 min at room temperature. For all reactions 20 mL steel jars and two stainless steel balls of 7 mm diameter were used.

**HAL**^**+**^**·MO**^**–**^, **HAL**^**+**^**·ME**^**–**^, **(HAL**^**+**^**)**_**2**_**·(FU**^**2–**^**)·(FU)**, **HAL**^**+**^**·AD**^**–**^ and **HAL**^**+**^**·PI**^**–**^ were obtained using a 1:1 mixture of HAL (0.5 mmol, 187.95 mg) and the respective coformer (0.5 mmol, 52.03 mg of MO, 58.03 mg of ME and FU, 73.07 mg of AD and 80.08 mg of PI) and 100 μL of ethyl acetate.

**HAL**^**+**^**·GU**^**–**^ was obtained using a 1:1 mixture of HAL (0.5 mmol, 187.95 mg), GU (0.5 mmol, 66.06 mg) and 100 μL of benzene.

**(HAL**^**+**^**)**_**2**_**·(TR**^**2–**^**)·(TR)**, **(HAL**^**+**^**)**_**2**_**·(SU**^**2–**^**)·(SU)** were obtained using a 1:1 mixture of HAL (0.5 mmol, 187.95 mg) and the respective coformer (0.5 mmol, 83.03 mg of TR and 87.10 mg of SU), and 50 μL of H_2_O.

Powder X-ray diffraction (PXRD) was used to analyze all products obtained and determine the formation of a new PMM, as well as their crystallinity and purity. Furthermore, all operations were repeated by triplicate to ensure reproducibility.

#### 2.1.2 Preparation of Single Crystals

Single crystals of the novel HAL phases were obtained after slow evaporation (1 day, 20 °C) of saturated solutions prepared by dissolving 10–20 mg of the product of the LAG in 500 μL of the same solvent used for the mechanochemical synthesis. To eliminate the excess of nondissolved product, the solutions were filtered using through 0.22 μm syringe filters. Suitable crystals for single-crystal X-ray diffraction (SCXRD) analysis and structure determination were carefully isolated from the solution.

### 2.2 Characterization Techniques

#### 2.2.1 X-ray Diffraction Analysis

PXRD analyses were performed at room temperature on a Bruker D8 Advance Vαrio diffractometer (Bruker-AXS, Karlsruhe, Germany) equipped with a LYNXEYE detector and CuK_α1_ radiation (1.5406 Å). The diffractograms were recorded over an angular range of 5–50° (2θ) with a step size of 0.02° (2θ) and a total measurement time of 30 min.

SCXRD data were obtained at room temperature on a Bruker D8 Venture diffractometer (Bruker-AXS, Karlsruhe, Germany) using CuKα radiation (λ = 1.54178 Å). The data were processed with the APEX4 suite.^27^ The structure was solved with intrinsic phasing^28^ and refined with full-matrix least-squares on *F*^2^(29) using Olex2 as a graphical interface.^30^ The non-hydrogen atoms were refined anisotropically. Hydrogen atoms were located in difference Fourier maps and included as fixed contributions riding on attached atoms with isotropic thermal displacement parameters 1.2 or 1.5 times those of the respective atom. for the analysis and visualization of the structure and also for graphic material preparation Mercury^31^ software was used. CIF files are deposited in the Cambridge Structural Database (CSD) under the CCDC numbers: 2054669–2054676. Copies of the data can be obtained free of charge at https://www.ccdc.cam.ac.uk/structures/.

#### 2.2.2 DFT Calculations

The calculations of noncovalent interactions were carried out using Gaussian-16 at the PBE1PBE-D3/def2-TZVP level of theory. The Grimme’s D3 dispersion correction has been used in the calculations^32^ since it is adequate for the correct evaluations of π-stacking interactions. To gain insight into the noncovalent interactions observed in the solid state, we performed DFT calculations on molecular clusters extracted from the crystallographic structures. These finite assemblies reproduce the key intermolecular interactions identified in the crystal lattice, particularly hydrogen bonding and other directional contacts. The geometries were taken directly from the X-ray structures, and only the hydrogen positions involved in H-bonds were optimized to better reflect realistic interaction. Geometries. This procedure and level of theory has been used before to investigate similar interactions in the solid state.^33,34^ The interaction energies were computed by calculating the difference between the energies of the isolated monomers and the ones of their assembly. The QTAIM analysis^35^ and NCIplot index^36^ have been computed at the same level of theory by means of the AIMAll program.^37^

#### 2.2.3 Differential Scanning Calorimetry

Simultaneous Differential scanning calorimetry (DSC) and Thermogravimetric (TGA) studies were performed in a NETZSCH STA 449 F5 calorimeter (NETZSCH Group, Germany). Experimental conditions: alumina (Al_2_O_3_) crucibles of 85 μL volume, atmosphere of dry nitrogen with 250 mL/min flow rate, and heating rates of 5 °C/min ranging from 25 to 250 °C. The calorimeter was calibrated with indium of 99.99% purity (m.p.: 156.4 °C; DH: 28.14 J/g).

#### 2.2.4 Thermodynamic Stability Analysis

The thermodynamic stability of the solid forms was evaluated under accelerated aging conditions. For that purpose, 200 mg of the new solids were placed in watch glasses and left at 40 °C and 75% RH in a Memmert HPP110 climate chamber (Memmert, Schwabach, Germany). The integrity under the above-mentioned conditions was periodically monitored using PXRD for 3 months.

Additionally, the stability in aqueous suspension was evaluated through slurry experiments in which an excess of powder sample was added to 0.5 mL of (1) buffer using buffer KCl (0.2 M) pH 1.2, prepared by dissolving 14.91 g of KCl in 1000 mL of MiliQ-water, and (2) phosphate buffer solution (PBS) (0.2 M) pH 6.8, prepared by dissolving 27.22 g of KH_2_PO_4_ in 1000 mL of MiliQ-water. The pH of both buffers was adjusted using HCl 0.1 M and NaOH 0.1 M solutions. After 24 h of stirring at 25 °C in sealed vials, the solids were collected, filtered, dried, and analyzed with PXRD.

#### 2.2.5 Solubility Studies

Solubility studies were carried out following the shake-flask method^38^ using buffer KCl (0.2 M) pH 1.2, and buffer PBS (0.2 M) pH 6.8. In these experiments, saturated solutions of HAL and the novel HAL-PMMs were prepared by adding an excess of solid to 10 mL of each buffer. After 24 h of stirring at 25 °C the solutions were filtered through 0.22 μm syringe filters, diluted to achieve a measurable concentration and directly measured using high-performance liquid chromatography (HPLC). Samples were evaluated at measured at 248 nm, HAL maximum of absorbance. HPLC experiments were performed with an Agilent 1200 HPLC system (Agilent Technologies, Santa Clara, CA, USA) equipped with a solvent degasser, pump, autosampler, and diode array detector. A Scharlau (Barcelona, Spain) 100 C18 chromatographic column (3 μm, 150 × 4.6 mm) was set at 25 °C and used for compound separation, using an isocratic elution method. The mobile phase was composed of a mixture of 90% acetonitrile and 10% water. The flow rate was 0.8 mL/min, and the injection volume was 30 μL. ChemStation software was used for data acquisition and analysis (Agilent Technologies, Santa Clara, CA, USA). The retention time for HAL was 5 min 45 s in KCl and 6 min 55 s in PBS, and the concentration for the calibration curve was determined from the area under the HAL peak.

## 3 Results and Discussion

### 3.1 *In Silico* Coformer Screening

A preliminary *in silico* coformer screening was previously conducted to enhance the success rate of mechanochemical synthesis. A Cambridge Structural Database (CSD) survey identified 34 entries, including HAL base (REFCODE: HALDOL) and its salts: chloride (BIDFUQ), bromide (HALOPB), saccharinate (YANMUW), and picrate (CUCYUV). HAL salts with ME,^39^ salicylic acid, hydroxybenzoic acids (3,4-, 3,5-, and 4-hydroxybenzoic acid)^40^ as well as hydrates with oxalic, succinic, and FU acids^41^ were also reported. In all these cases, salt formation is evident due to proton transfer from the acid coformer to the piperidine nitrogen of HAL, reflecting the basic nature of HAL (p*K*_a_ = 8.30) and its tendency to form salts and hydrates, which confirms that dicarboxylic acids can be used as counterions for pharmaceutical salts of HAL.

The coformer selection was also validated using the COSMOQuick software.^42^ This software estimates the excess enthalpy of formation (*H*_ex_) between HAL and each coformer, relative to the pure components in their supercooled liquid states.^42^ Compounds with negative *H*_ex_ values are more likely to form new multicomponent materials, as *H*_ex_ approximates the free energy of cocrystal formation (Δ*G*_cocrystal_). Moreover, conformational flexibility is accounted for by means of an empirical parameter, *f*_fit_, which incorporates both Δ*H*_ex_ and the number of rotatable bonds present in the compounds. The results of this analysis, shown in Table 1, qualitatively confirm that dicarboxylic acids can be used as counterion for pharmaceutical salts of HAL but, interestingly, *H*_ex_ values close to 0 and quite high *f*_fit_ values are obtained with aliphatic long acids. We hypothesized that this reduced tendency can be attributed to the steric impediments in the conformation and the higher molecular flexibility of the coformer, which allows them to adopt multiple arrangements, potentially reducing the specificity and strength of interactions with HAL. Additionally, their larger hydrophobic regions further limit the formation of strong hydrogen-bonding networks necessary for stable multicomponent material formation.

**Table 1:** COSMOQuick Calculations of *H*_ex and_*f*_fit_ for the Selection of Coformers

coformer	*H*_ex_ (kcal/mol)	*f*_fit_
Fumaric Acid	–1.40029	4.212
Maleic acid	–1.22035	4.392
Terephthalic acid	–1.219475	4.393
Glutaric acid	–0.428955	6.204
Adipic Acid	–0.44068	6.702
Pimelic Acid	–0.45906	7.194
Suberic Acid	–0.231015	7.932

### 3.2 Mechanochemical Synthesis

**Figure 2:**
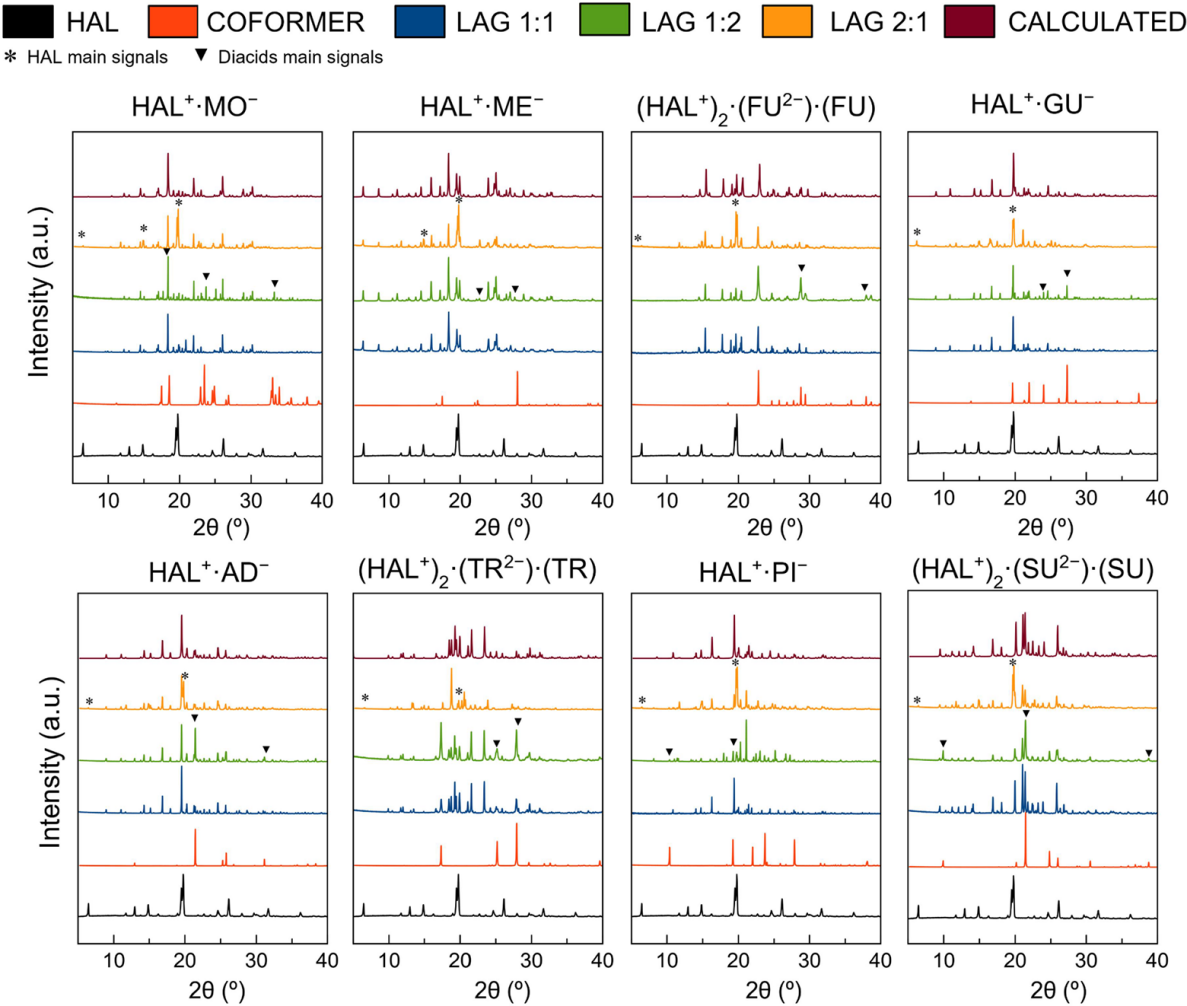
PXRD patterns of the novel HAL phases at different stoichiometries, along with the calculated PXRD pattern obtained from SCXRD data.

The new multicomponent materials of HAL were obtained via liquid-assisted grinding (LAG), a widely applied method for the screening and synthesis of multicomponent materials due to their time and resource consumption efficiency.^43^ Initial screenings involved 1:1, 1:2, and 2:1 reactions, in which new materials emerged. However, an excess of one of the initial components (either HAL or the respective coformer) was observed when 1:2 and 2:1 stoichiometries were used, indicating that the correct stoichiometric approach was 1:1 for all the novel phases, as shown in Figure 2. These results underline the importance of analyzing mixtures with different stoichiometries to identify the correct stoichiometry of the novel materials.

### 3.3 Crystal Structure Analysis

Despite the promising results of LAG reactions, the crystal structure of the multicomponent materials is required to confirm the formation of a pharmaceutical salt and discard the formation of neutral cocrystals, polymorphs or hydrates/solvates. Moreover, the crystal structure is necessary to explain and establish relationships with the final physicochemical properties of the new materials. For this purpose, good-quality single crystals were obtained after 1 day of evaporation of saturated solutions of the LAG product. The crystal structure also provides a calculated powder pattern of the new phases, which can be compared with the PXRD patterns obtained by LAG. The good agreement of the calculated and experimental PXRD patterns confirms the phase purity of the bulk product (Figure 2) allowing its use for further characterization.

Crystal structure determination via single-crystal X-ray diffraction (SCXRD) revealed the formation of two different types of pharmaceutical multicomponent materials, salts and ionic cocrystals. In general, salt formation in molecular crystals is considered when the Δp*K*_a_ between the acid and base components exceeds 3, while zero or negative Δp*K*_a_ values consistently result in cocrystal formation. This principle, known as the “Δp*K*_a_ rule”, has been widely applied in the design of salts and cocrystals, facilitated by the availability of both experimental and computational p*K*_a_ data.^44,45^ In 2012, Cruz-Cabeza refined this rule after analyzing approximately 6,500 crystal structures and their calculated p*K*_a_ values. The study found that Δp*K*_a_ > 4 almost always leads to salt formation, whereas Δp*K*_a_ < −1 strongly favors cocrystals. For intermediate values (−1 < Δp*K*_a_ < 4), the degree of ionization must be deeply studied, as depends on the whole crystal packing, including secondary interactions or the size of the coformer.^46,47^ In fact, the length of the coformer molecule has an inverse relation with the acidity (p*K*_a_) as shown in Table 2.

**Table 2:** Acidity Values (p*K*_a_) of the Molecules and the Difference of Acidity (Δp*K*_a1_) of the Coformers with HAL

molecule	p*K*_a1_	p*K*_a2_	Δp*K*_a1_	ref
Haloperidol	8.05	13.96		(48−50)
Maleic acid	1.94	6.22	6.11	(51−53)
Malonic acid	2.85	5.05	5.20	(52,53)
Fumaric acid	3.03	4.54	5.02	(54,55)
Terephthalic acid	3.51	4.82	4.54	(52,56)
Glutaric acid	4.34	5.41	3.71	(52,57)
Adipic acid	4.41	5.41	3.64	(58,59)
Pimelic acid	4.50	5.43	3.55	(60,61)
Suberic acid	4.53	5.50	3.52	(62)

Following this premise, the formation of ionic species of HAL with small-strong acids such as MO and ME is an expected result due to the differences in acidity (Table 2)**.** However, in the other cases, the Δp*K*_a_ ranges from 3.50 to 5, allowing for the obtention of intermediate species between salts and cocrystals. The analyses by SCXRD finally determined that HAL formed salts in a 1:1 stoichiometry with dicarboxylic acids ranging from C3 to C7, (MO, ME, GU, AD and PI) while 1:1 ionic cocrystals were obtained with FU, TR and SU acids.

**Figure 3:**
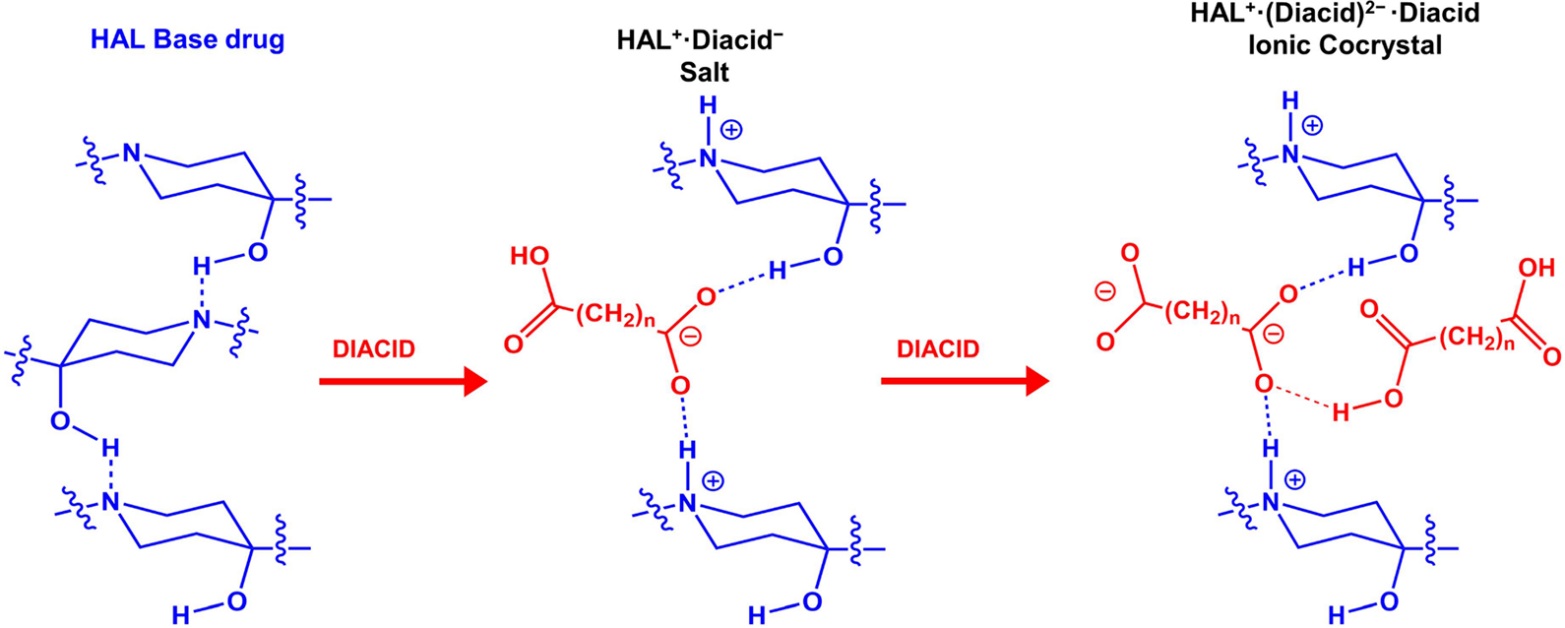
Schematic representation of the formation of the salts and ionic cocrystals of HAL.

Previous studies have reported the native structure of HAL in which HAL molecules forms chains through hydrogen bonds involving its amino and hydroxyl groups.^63,64^ During salt formation, protonation of HAL and the presence of the counterion lead to the integration of the acid into the chain, which separates HAL^+^ molecules through electrostatic hydrogen bonds involving the −COO^–^ of the acid and the −NH^+^ of HAL (Figure 3). This finding is further supported by the experimental electron density map (Figure S1) and validated through analysis of the C–O bond distances in the carboxylate group of the acids (Table S1), with Δ*D*_C–O_ values closely matching those observed in salts, ranging from 0.008 to 0.024 Å.^65^

**Figure 4:**
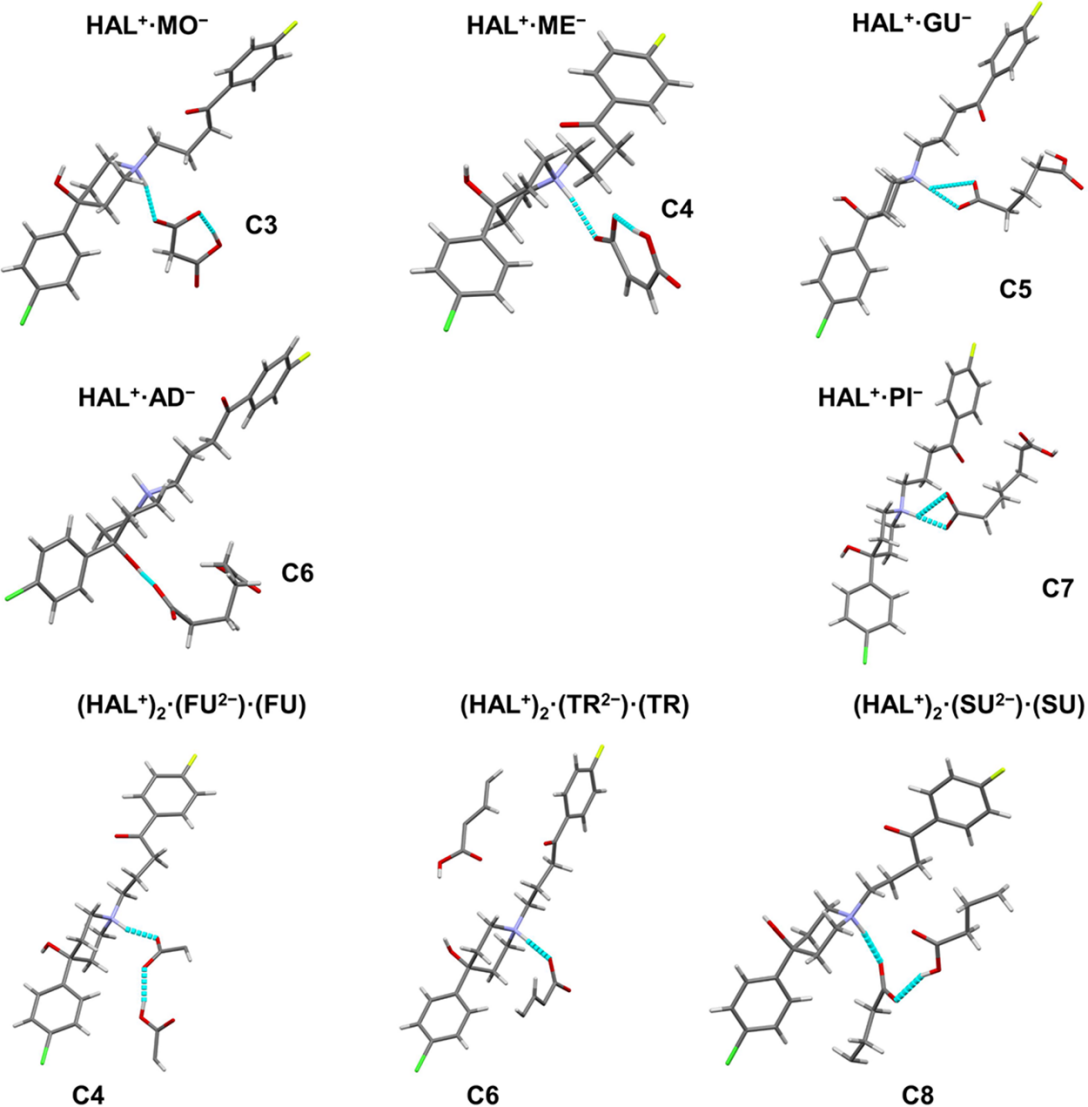
Asymmetric units of the novel reported phases of HAL in this work.

While MO, ME, GU, AD and PI coformers lead to the corresponding HAL^+^·Acid**^–^** salts, a different result is observed for FU, TR and SU coformers, which yield ionic cocrystals. In these cases, an ionic acid molecule connects two HAL^+^ molecules via **–**N**–**H^+^···O^**–**^**–**OC**–** hydrogen bond. As shown in Figure 3, to equilibrate the charge in these materials, the molecule of diacid that connects the two HAL^+^ molecules is twice deprotonated. In other words, the cocrystallization of the previously formed HAL^+^·Acid**^–^** salt with a neutral acid molecule occurs leading to the formation of an ionic cocrystal. Considering the Δp*K*_a_ rule and the data presented in Table 2, the Δp*K*_a_ of MO and ME leads to a direct ionization of the molecules, however the other coformers present Δp*K*_a_ ranging from 3 to 5, which depending on the crystal packing, can lead to the inclusion of neutral molecules in the final structure, giving rise to ionic cocrystals. In fact, the second p*K*_a_ of FU and TR are the lowest, so they have a higher tendency to deprotonate twice, allowing the above-mentioned ionic cocrystal arrangement. The crystallographic information of the new solids of HAL is gathered in Table 3 at the end of this section, while the asymmetric units are represented in Figure 4.

**Table 3:** Crystallographic Data of HAL and the Reported PMMs

compound name	HAL^+^·MO^–^	HAL^+^·ME^–^	(HAL^+^)_2_·(FU^2–^)·(FU)
CCDC number	2054669	2054670	2054671
Formula	C_24_H_27_ClFNO_6_	C_25_H_27_ClFNO_6_	C_25_H_27_ClFNO_6_
Molecular weight	479.91	491.92	491.92
Crystal system	monoclinic	monoclinic	monoclinic
Space group	*P*2_1_/*n* (14)	*C*2/*c* (15)	*P*2_1_/*n* (14)
*a* [Å]	15.0579(11)	28.035(13)	13.9564(11)
*b* [Å]	10.1677(6)	11.106(5)	13.8190(12)
*c* [Å]	15.7274(11)	16.055(6)	14.2403(12)
α [°]	90	90	90
β [°]	106.032(2)	99.686(14)	118.285(3)
γ [°]	90	90	90
Volume [Å^3^]	2314.3(3)	4928(4)	2418.5(4)
*Z*	4	8	4
ρ_calc_ [g cm^–3^]	1.377	1.326	1.351
*F*(000)	1008	2064	1032
Reflections collected	43,605	22,021	19,241
Unique reflections	5313 [*R*_int_ = 0.0994]	4331 [*R*_int_ = 0.0987]	4123 [*R*_int_ = 0.1768]
Data/Restraints/Parameters	5313/1/303	4331/0/309	4123/0/310
Goodness-of-fit on *F*^2^	1.031	1.063	1.014
Final *R* indexes	*R*_1_ = 0.0511	*R*_1_ = 0.0776	*R*_1_ = 0.0649
[*I* ≥ 2σ(*I*)]	*wR*_2_ = 0.1128	*wR*_2_ = 0.2003	*wR*_2_ = 0.0966

compound name	**HAL**^**+**^**·GU**^**–**^	**HAL**^**+**^**·AD**^**–**^	**(HAL**^**+**^**)**_**2**_**·(TR**^**2–**^**)·(TR)**
CCDC number	2054672	2054673	2054674
Formula	C_26_H_31_ClFNO_6_	C_27_H_33_ClFNO_6_	C_29_H_29_ClFNO_6_
Molecular weight	507.96	521.99	541.98
Crystal system	monoclinic	monoclinic	triclinic
Space group	*Cc* (9)	*Cc* (9)	*P*1̅ (2)
*a*/Å	23.276(5)	23.513(2)	8.2356(2)
*b*/Å	8.8655(18)	8.7433(7)	9.6967(3)
*c*/Å	14.643(3)	15.2632(14)	18.4015(5)
α/°	90	90	92.063(2)
β/°	121.814(6)	122.902(3)	102.661(2)
γ/°	90	90	112.541(2)
*V*/Å^3^	2567.7(10)	2634.5(4)	1312.45(7)
*Z*	4	4	2
*D*_c_/g cm^–3^	1.311	1.316	1.371
*F*(000)	1072	1104	568
Reflections collected	25,581	17,003	17,614
Unique reflections	4507 [*R*_int_ = 0.0660]	4380 [*R*_int_ = 0.0514]	4572 [*R*_int_ = 0.0420]
Data/Restraints/Parameters	4507/2/318	4380/2/327	4572/0/346
Goodness-of-fit on *F*^2^	1.066	1.033	1.031
Final *R* indexes	*R*_1_ = 0.0499	*R*_1_ = 0.0410	*R*_1_ = 0.0555
[*I* ≥ 2σ(*I*)]	*wR*_2_ = 0.1095	*wR*_2_ = 0.0949	*wR*_2_ = 0.1368

compound name	**HAL**^**+**^**·PI**^**–**^	**(HAL**^**+**^**)**_**2**_**·(SU**^**2–**^**)·(SU)**
CCDC number	2054675	2054676
Formula	C_28_H_35_ClFNO_6_	C_29_H_37_ClFNO_6_
Molecular weight	536.02	550.04
Crystal system	monoclinic	triclinic
Space group	*Cc* (9)	*P*1̅ (2)
*a* [Å]	24.3284(16)	8.678(4)
*b* [Å]	8.8560(5)	8.917(4)
*c* [Å]	15.0616(10)	18.749(8)
α [°]	90	87.286(14)
β [°]	122.263(4)	83.064(15)
γ [°]	90	73.961(13)
Volume [Å^3^]	2744.0(3)	1384.0(11)
*Z*	4	2
ρ_calc_ [g cm^–3^]	1.297	1.320
*F*(000)	1136	584
Reflections collected	25,782	28,532
Unique reflections	4807 [*R*_int_ = 0.0579]	4855 [*R*_int_ = 0.0506]
Data/Restraints/Parameters	4807/2/336	4855/0/346
Goodness-of-fit on *F*^2^	1.085	1.044
Final *R* indexes	*R*_1_ = 0.0746	*R*_1_ = 0.0432
[*I* ≥ 2σ(*I*)]	*wR*_2_ = 0.2100	*wR*_2_ = 0.1115

#### 3.3.1 **HAL^+^·MO^–^**and **HAL^+^·ME^–^** Salts

**Figure 5:**
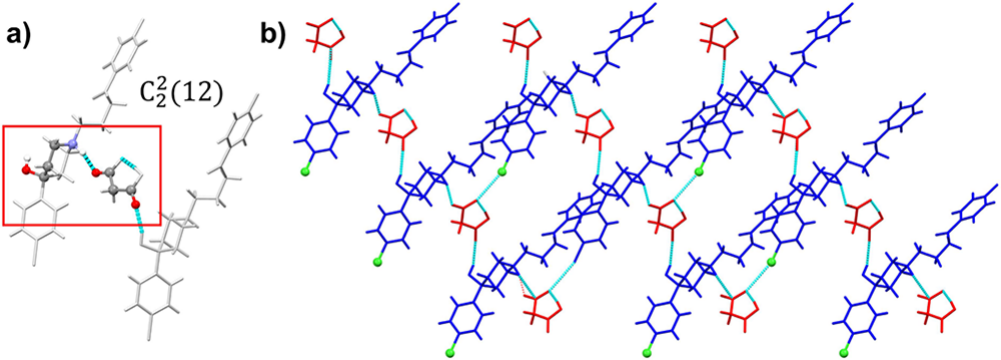
(a) Graph set of **HAL**^**+**^**·MO**^**–**^**.** HAL^+^ cations are represented as sticks and MO^–^ as balls and sticks. (b) Detailed view of the layered structure in **HAL**^**+**^**·MO**^**–**^. HAL^+^ cations are represented in blue and MO^–^ in red.

**HAL**^**+**^**·MO**^**–**^ and **HAL**^**+**^**·ME**^**–**^ present asymmetric units containing one molecule of HAL and one molecule of the acid, both of them in the ionic state (Figure 4). These salts crystallize in the monoclinic system with *P*2_1_/*n* space group for **HAL**^**+**^**·MO**^**–**^ and *C*2/*c* for **HAL**^**+**^**·ME**^**–**^. Despite differing in their space groups, these phases exhibit similar hydrogen bonding interactions between HAL^+^ and the acid, characterized by a pattern designator of type C (chain)^66,67^ with graph sets *C*_2_^2^(12) and *C*_2_^2^(13) for **HAL**^**+**^**·MO**^**–**^ and **HAL**^**+**^**·ME**^**–**^, respectively (Figure 5a). This arrangement allows the coformer molecule to connect two molecules of HAL^+^ via N**–**H^+^···**^–^**OOC and COOH···OH hydrogen bonds. The two carboxyl groups of the coformer are involved, stabilizing the structure through an additional intramolecular hydrogen bond within the coformer and a discrete F···O interaction with a third molecule of HAL^+^. As shown in Figure 5b coformer molecules adopt a perpendicular orientation relative to HAL^+^ molecules, increasing spacing, which is associated with an enhancement of solubility in pharmaceutical multicomponent materials.^68−71^ These interactions lead to 2D hydrogen-bonded networks creating layers of HAL^+^ that are separated by layers of coformers (Figure 5b). This configuration extends into a 3D macromolecular structure through additional noncovalent interactions, including van der Waals forces and π···π stacking of the aromatic rings of HAL.

#### 3.3.2 **HAL^+^·GU^–^**, **HAL^+^·AD^–^**, and **HAL^+^·PI^–^** Salts

**Figure 6:**
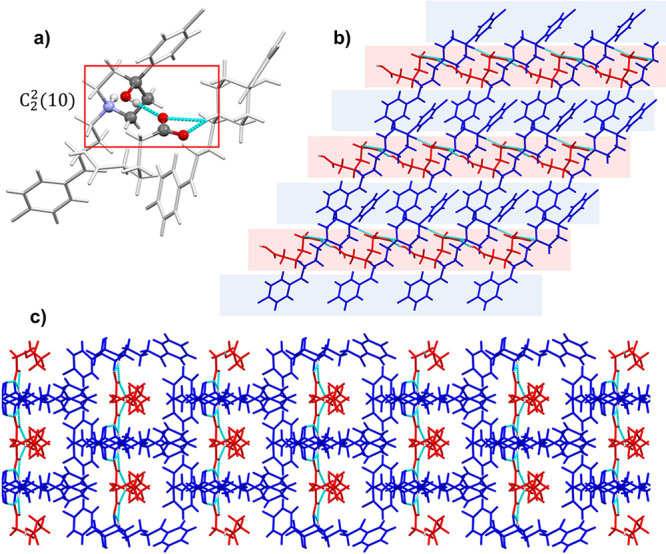
(a) Graph set of **HAL**^**+**^**·AD**^**–**^**.** HAL^+^ cations are represented as sticks and AD^–^ as balls and sticks. (b) Detailed view of the layered structure and (c) 3D structure observed in **HAL**^**+**^**·AD**^**–**^. HAL^+^ cations are represented in blue and AD^–^ in red.

**HAL**^**+**^**·GU**^**–**^**, HAL**^**+**^**·AD**^**–**^ and **HAL**^**+**^**·PI**^**–**^ salts also present asymmetric units containing one cation of HAL^+^ and one acid anion (Figure 4). Similar crystallographic features are observed in these salts, which crystallize also in the monoclinic system but with *Cc* space group. Figure 6 shows the crystal structure of **HAL**^**+**^**·AD**^**–**^ as a representative case. In the crystal structure, two molecules of HAL^+^ are linked via N^+^–H···^–^OOC and O–H···^–^OOC hydrogen bonds described by the graph set *C*_2_^2^(10).^66,67^ In this configuration, the coformer bridges two HAL^+^ molecules through its ionic carboxylate group (Figure 6a), while the second–COOH remains available for interactions with adjacent coformer molecules via–COOH···^–^OOC–. The hydrogen bond network resulting from these interactions provide enhanced structural stability when compared with the **F···O** interactions observed in **HAL**^**+**^**·MO**^**–**^ and **HAL**^**+**^**·ME**^**–**^. One might assume that longer coformer molecules would increase the spacing between HAL^+^ molecules. However, in these salts, the length of the coformer does not allow a perpendicular disposition against HAL^+^ molecules. Consequently, this new arrangement leads to the formation of one-dimensional chains of coformer parallel to one-dimensional chains of HAL**^+^**, in which HAL^+^ molecules remain relatively close together, minimizing structural disruption compared to **HAL**^**+**^**·MO**^**–**^ and **HAL**^**+**^**·ME**^**–**^.

These chains further assemble into 2D networks that are stabilized by C–H···O interactions between HAL^+^ and the carboxylic groups of the anion, and additional π···π stacking interactions involving the aromatic rings of HAL^+^ molecules (Figure 6b). Stacking of these 2D networks forms the final 3D crystal lattice in which HAL^+^ chains surround the dicarboxylic acid anions (Figure 6c).

#### 3.3.3 **(HAL^+^)_2_·(FU^2–^)·(FU)**,**(HAL^+^)_2_·(TR^2–^)·(TR)**, **(HAL^+^)_2_·(SU^2–^)·(SU)** Ionic Cocrystals

The cases of **(HAL**^**+**^**)**_**2**_**·(FU**^**2–**^**)·(FU)** (C4) and **(HAL**^**+**^**)**_**2**_**·(TR**^**2–**^**)·(TR)** (C6) are particularly interesting because, even though the ΔpK_*a*_ difference between the components is greater than 4, suggesting that salt formation should be favorable, as observed with ME (C4) and AD (C6), ionic cocrystals were obtained instead. Understanding and identifying alternative factors that promote salt formation instead of ionic cocrystals is crucial for predicting and controlling the final solid-state properties.

ME (*cis*-isomer) present both carboxyl groups on the same side of the double bond, giving as a result an intramolecular hydrogen bond, which stabilizes the monoanionic form and facilitates the deprotonation of the first −COOH group, promoting the formation of salts. Conversely, FU (*trans*-isomer) present the carboxyl groups on opposite sides of the double bond, allowing for double deprotonation, and interacting with two molecules of HAL. On the other hand, TR is more favorable for double deprotonation than AD due to **resonance stabilization** and the **electron-withdrawing effect** of its benzene ring. This delocalizes the negative charge, making both deprotonations easier and ionic cocrystal formation favorable. In contrast, since AD lacks an aromatic system, it cannot stabilize the dianion effectively, making the second deprotonation less favorable, thus resulting in salt formation.

**Figure 7:**
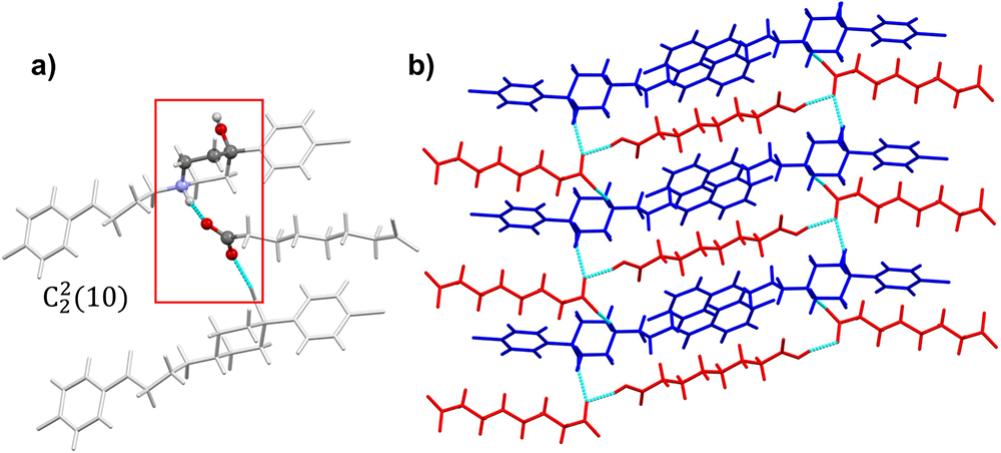
(a) Graph set of **(HAL**^**+**^**)**_**2**_**·(SU**^**2–**^**)·(SU)**. HAL^+^ cations are represented as sticks and SU^–^ as balls and sticks. (b) Detailed view of the layered structure observed in **(HAL**^**+**^**)**_**2**_**·(SU**^**2–**^**)·(SU)**. HAL^+^ cations are represented in blue and SU^–^ in red.

SCXRD analysis revealed that the asymmetric units of the ionic cocrystals (Figure 4) consist of one molecule of HAL^+^, 1/2 anionic acid molecule, and 1/2 additional neutral molecule of acid. Despite the presence of neutral molecules of coformers, the crystallographic features of these ionic cocrystals are similar to those of **HAL**^**+**^**·GU**^**–**^**, HAL**^**+**^**·AD**^**–**^ and **HAL**^**+**^**·PI**^**–**^ salts. In this case, **(HAL**^**+**^**)**_**2**_**·(FU**^**2–**^**)·(FU)**crystallizes in the monoclinic system, with *P*2_1_/*n* space group, while **(HAL**^**+**^**)**_**2**_**·(TR**^**2–**^**)·(TR)** and **(HAL**^**+**^**)**_**2**_**·(SU**^**2–**^**)·(SU)** crystallize in the triclinic system with P1̅ space group. Figure 7 illustrates the crystal structure of **(HAL**^**+**^**)**_**2**_**·(SU**^**2–**^**)·(SU)** as a representative case. The crystal structure of the ionic cocrystals presents interconnected HAL^+^ molecules bridged by dicarboxylic acids through via N^+^–H···^–^OOC and O–H···^–^OOC hydrogen bonds, as defined by the *C*_2_^2^(10) graph set^66,67^ (Figure 7a), following the same arrangement of the previously described **HAL**^**+**^**·GU**^**–**^**, HAL**^**+**^**·AD**^**–**^ and **HAL**^**+**^**·PI**^**–**^ salts. The mentioned interactions induce the formation of 1D chains of HAL^+^, separated by 1D chains of coformers, formed by ionic molecules intercalated with neutral ones. This structure is extended laterally, giving rise to 2D frameworks, which are further stabilized by interactions such as C–H···O bonds between the HAL^+^ and the carboxylate groups of the coformer (Figure 7b). Additionally, π···π stacking among the aromatic rings of HAL^+^ reinforces the structural cohesion. Stacked layers of these 2D networks culminate in a robust 3D crystal structure where HAL^+^ molecules surround the acid anions.

### 3.4 DFT Calculations

In addition to the crystal structure study, the theoretical analysis study of some unconventional noncovalent interactions observed in the solid state of the here-reported HAL PMMs can clarify the differences between phases, and could be useful to understand the differences in the physicochemical properties. These theoretical studies will be performed using DFT calculations (PBE1PBE-D3/def2-TZVP) combined with the quantum theory of atoms-in-molecules (QTAIM) and noncovalent interaction plot (NCIplot) index computational tools. In particular, this study focuses on the characterization of σ-hole halogen bonding (Cl-atom acts as Lewis acid) and π-hole tetrel bonding (C atom acts as Lewis acid) using QTAIM/NCIPlot analysis. In addition, more conventional H-bonding interactions have been also analyzed and their dissociation energies computed using the QTAIM method, as commented below.

**Figure 8:**
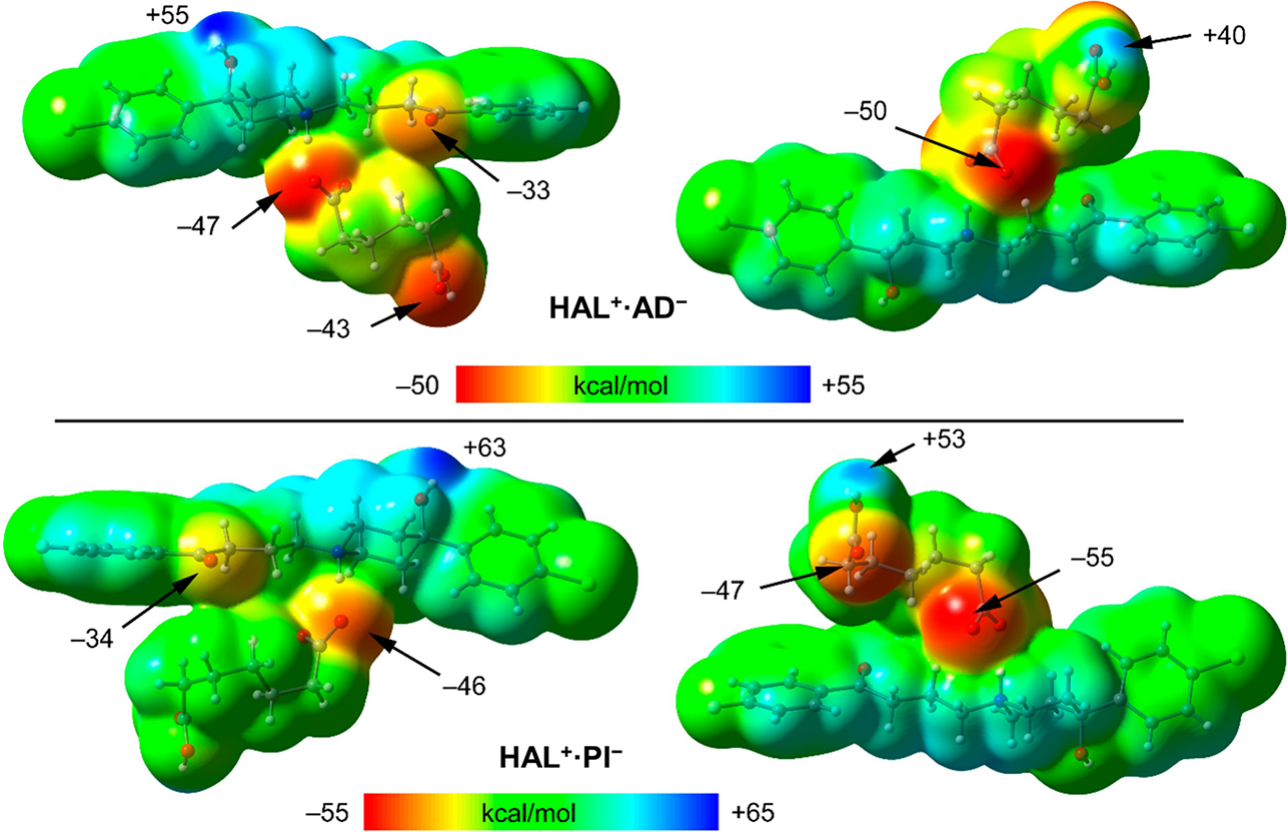
MEP surfaces (0.001 a.u. isosurface) of compounds **HAL**^**+**^**·AD**^**–**^ (top) and **HAL**^**+**^**·PI**^**–**^ (bottom) using two orientations. The values at selected points of the surfaces are given in kcal/mol (PBE0-D3/def2-TZVP).

First, we have computed the molecular electrostatic potential (MEP) surfaces of some representative haloperidol salts to investigate the most electron-rich and electron-poor regions within the crystalline salt assemblies. Figure 8 shows the surfaces of compounds **HAL**^**+**^**·AD**^**–**^ and **HAL**^**+**^**·PI**^**–**^ using two orientations. In both salts, the most positive MEP values are observed at the hydrogen atom of the hydroxyl group, followed by the hydrogen atom of the carboxylic acid group (+55 and +66 kcal/mol for **HAL**^**+**^**·AD**^**–**^ and **HAL**^**+**^**·PI**^**–**^, respectively). This distribution reflects the localization of these functional groups on the cationic (HAL^+^) and anionic (carboxylate) components of the salts, which governs the observed electrostatic potential. The most negative MEP is located at the O atom of the carboxylate group in both compounds followed by the O atom at the carboxy group. The MEP values at the carbonyl O atoms of the haloperidol molecules are also negative (−33 and −34 kcal/mol for **HAL**^**+**^**·AD**^**–**^ and **HAL**^**+**^**·PI**^**–**^, respectively).

**Figure 9:**
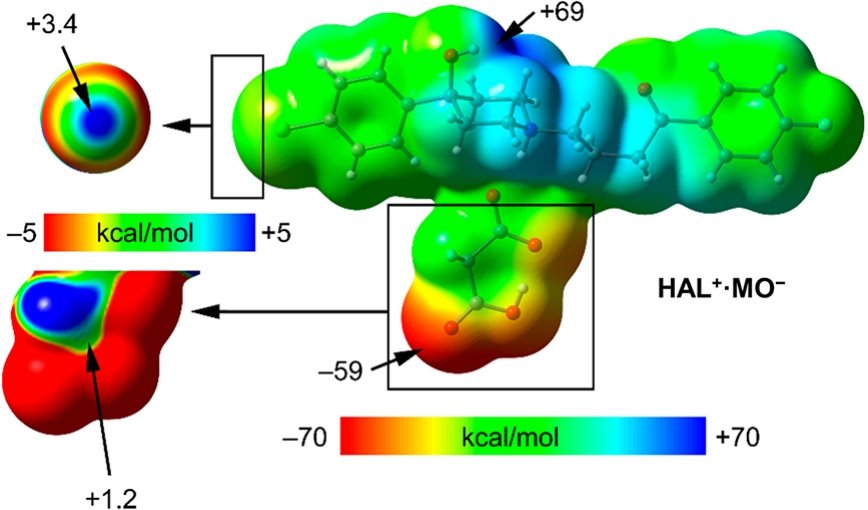
MEP surface (0.001 au isosurface) of compound **HAL**^**+**^**·MO**^**–**^ and its amplification around Cl and malonate moieties used a reduced scale (±5 kcal/mol). The values at selected points of the surfaces are given in kcal/mol (PBE1PBE-D3/def2-TZVP).

Figure 9 shows the MEP surface of compound and **HAL**^**+**^**·MO**^**–**^ where the crystal coformer is malonate. Similarly to compounds **HAL**^**+**^**·AD**^**–**^ and **HAL**^**+**^**·PI**^**–**^, the MEP maximum is located at the hydroxy H atom (+69 kcal/mol) and the MEP minimum at the O atom of the carboxy group (−59 kcal/mol). For this compound, we have examined the MEP surface around the chlorine atom of haloperidol to investigate the existence of a σ-hole on the extension of the C–Cl bond. To do so, we have used a reduced scale (see Figure 9, top-left) and the MEP surface discloses the existence of a very modest σ-hole (+3.4 kcal/mol) that is available for interacting with electron-rich atoms. We have also investigated the malonate anion in detail (see Figure 9, left) using a reduced scale, which reveals the existence of a positive region approximately located over the C atom of the carboxy group (+1.2 kcal/mol). To sum up, the MEP surfaces gathered in Figures 8 and 9 show the presence of strong H-bond donor and acceptor groups (−OH, −COOH and −COO^–^) and also reveal that the Cl and COOH groups can participate in weak interactions as electron acceptors via their σ- or π-hole, respectively.

**Figure 10:**
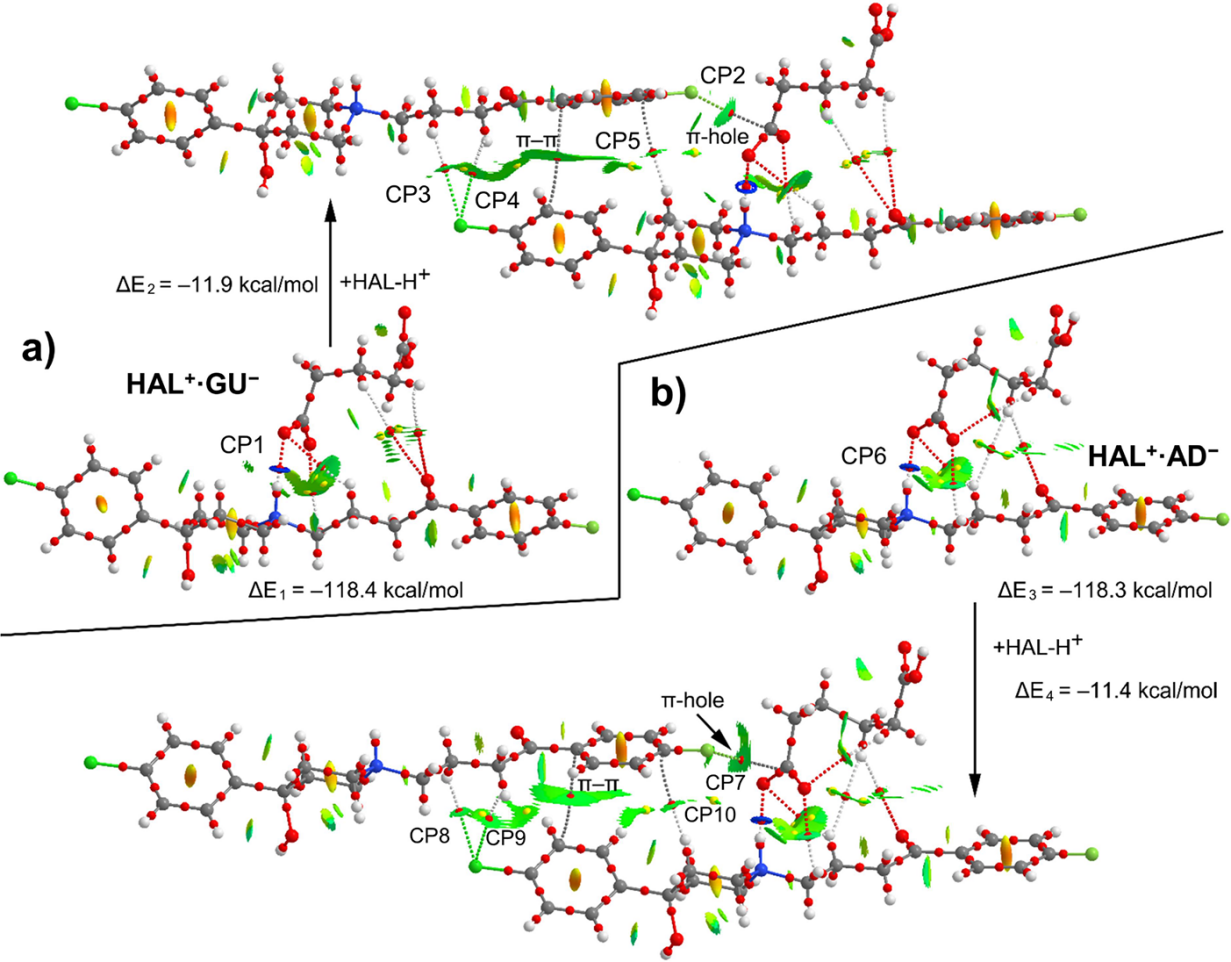
Combined QTAIM (bond and ring CPs in red and yellow, respectively and bond paths) and NCIplot analyses of the salts of compounds **HAL**^**+**^**·GU**^**–**^ (a) and **HAL**^**+**^**·AD**^**–**^ (b) and the interaction with the adjacent HAL^+^ molecule.

Figure 10 shows the QTAIM/NCIplot distribution of critical points (CPs) and bond paths of compounds **HAL**^**+**^**·GU**^**–**^ and **HAL**^**+**^**·AD**^**–**^, where the already observed via SCXRD electrostatically enhanced N^+^–H···^–^OOC H-bonds are formed. The QTAIM parameters of the CPs labeled in Figure 10 are summarized in Table 4, which also includes the dissociation energy of each H-bond estimated using the potential energy density (*V*_r_) at the bond CP.^72^ In fact, the interaction energies are very large and almost identical for both compounds (Δ*E*_1_ = −118.4 kcal/mol and Δ*E*_3_ = −118.3 kcal/mol) due to the strong electrostatic attraction. Both H-bonds are characterized by bond CPs and a dark blue isosurfaces (meaning strong attractive interaction) with concomitant dissociation energies of 14.4 and 14.2 kcal/mol (CP1 and CP6, see e) for **HAL**^**+**^**·GU**^**–**^ respectively, confirming its strong nature. Interestingly, in the solid state of both compounds the adjacent HAL molecule establishes a F···C contact with the C atom of the carboxylate group as evidenced by the QTAIM and NCIplot computational tools (see CP2 & CP7 in Figure 10). We have computed the interaction energies between **HAL**^**+**^**·GU**^**–**^ and **HAL**^**+**^**·AD**^**–**^ salts and adjacent HAL^+^ moieties, which are Δ*E*_2_ = −11.9 kcal/mol and Δ*E*_4_ = −11.3 kcal/mol, that correspond to the total contribution of several contacts in addition to the π-hole tetrel bonding (TtB), The π-hole F···C contact is expected to be extremely weak taking into consideration the MEP surface analysis commented above for **HAL**^**+**^**·AD**^**–**^. In both compounds, the QTAIM analysis shows the existence of two C–H···Cl H-bonds (CP3 & CP4 in **HAL**^**+**^**·GU**^**–**^ and CP8 & CP9 in **HAL**^**+**^**·AD**^**–**^ salts) and a C–H···π (CP5 in **HAL**^**+**^**·GU**^**–**^ and CP10 in **HAL**^**+**^**·AD**^**–**^) interaction. Moreover, it also reveals the existence of a π–π interaction characterized by a bond CP and a green isosurface in both compounds.

**Table 4:** QTAIM ρ_r_, *V*_r_, *G*_r_, and *H*_r_ Parameters at the Bond CPs Labelled in Figures 3–9 in a.u. and Predicted Dissociation Energies (kcal/mol) for Each Interaction Using the Electronic Potential Energy Densities [*E* = 0.5*V*_r_ for HBs and 0.556*V*_r_ for HaBs]

compound	CP	ρ_r_	*V*_r_	*G*_r_	*H*_r_	HB/*HaB* energy
**HAL**^**+**^**·GU**^**–**^	CP1	0.0459	–0.0432	0.0363	–0.0069	14.4
	CP2	0.0067	–0.0044	0.0061	0.0017	
	CP3	0.0060	–0.0029	0.0040	0.0011	0.91
	CP4	0.0043	–0.0022	0.0031	0.0009	0.69
	CP5	0.0034	–0.0016	0.0023	0.0007	
**HAL**^**+**^**·AD**^**–**^	CP6	0.0451	–0.0422	0.0359	–0.0063	14.2
	CP7	0.0056	–0.0027	0.0037	0.0010	
	CP8	0.0037	–0.0018	0.0027	0.0009	0.56
	CP9	0.0040	–0.0020	0.0027	0.0007	0.62
	CP10	0.0040	–0.0020	0.0027	0.0007	
**HAL**^**+**^**·MO**^**–**^	CP11	0.0405	–0.0468	0.0462	–0.0006	14.7
	CP12	0.0026	–0.0012	0.0017	0.0005	0.37
	CP13	0.0050	–0.0026	0.0037	0.0011	0.82
**HAL**^**+**^**·ME**^**–**^	CP14	0.0375	–0.0334	0.0315	–0.0019	10.5
	CP15	0.0084	–0.0052	0.0074	0.0022	1.81
**HAL**^**+**^**·PI**^**–**^	CP16	0.0498	–0.0487	0.0400	–0.0087	15.3
	CP17	0.0050	–0.0032	0.0047	0.0015	1.00
**(HAL**^**+**^**)**_**2**_**·(FU**^**2–**^**)·(FU)**	CP18	0.0463	–0.0445	0.0379	–0.0066	13.9
	CP19	0.0345	–0.0347	0.0359	0.0012	10.9
**(HAL**^**+**^**)**_**2**_**·(SU**^**2–**^**)·(SU)**	CP20	0.0394	–0.0368	0.0344	–0.0024	11.5
	CP21	0.0332	–0.0323	0.0333	0.0010	10.1
**(HAL**^**+**^**)**_**2**_**·(TR**^**2–**^**)·(TR)**	CP22	0.0489	–0.0501	0.0433	–0.0068	15.7
	CP23	0.0350	–0.0343	0.0344	0.0001	10.7

**Figure 11:**
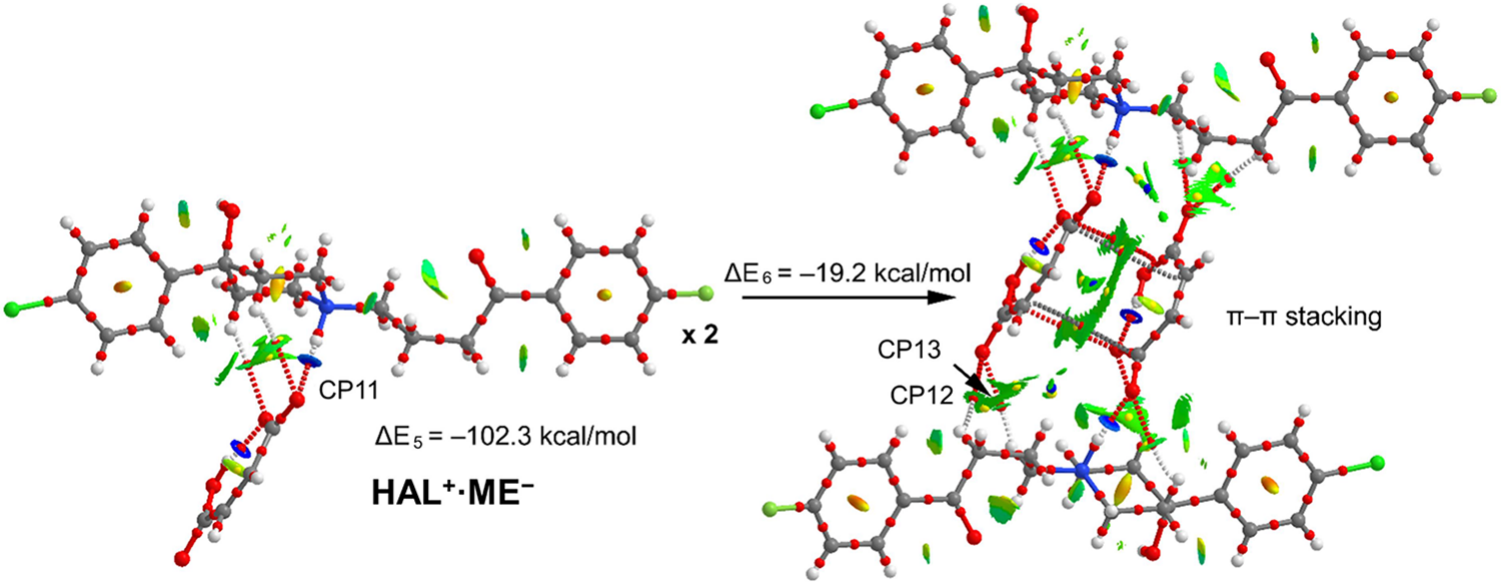
Combined QTAIM (bond and ring CPs in red and yellow, respectively and bond paths) and NCIplot analyses of **HAL**^**+**^**·ME**^**–**^ (left) and its dimer (right).

For compound **HAL**^**+**^**·ME**^**–**^, we have studied a remarkable and unique stacking interaction that is formed between the π-systems of two anions (see Figure 11). The planarity of maleate anion, the presence of a double bond and the intramolecular H-bond facilitates the stacking. The energetic results shown in Figure 11 reveal that the interaction energy of the salt is very large (Δ*E*_5_ = −102.3 kcal/mol) where in addition to the strong N^+^–H···^–^OOC H-bond (14.7 kcal/mol, see Table 4), two weak C–H···O are also formed, as confirmed by both the QTAIM and NCIplot analyses. The dimerization energy of **HAL**^**+**^**·ME**^**–**^ to yield the centro-symmetric supramolecular assembly is large (Δ*E*_6_ = −19.2 kcal/mol), thus revealing that this π-stacking motif is relevant in the solid state of **HAL**^**+**^**·ME**^**–**^. This quite unconventional π-stacking is characterized by four bond CPs that interconnect four atoms of each maleate anion and a green and extended green isosurface that confirms the large overlap of the π-systems. Upon dimerization, four symmetrically related C–H···O interactions are also established (see CP12 and CP13, in Figure 11). The contribution of these four H-bonds has been estimated using the *V*_r_ energy predictor resulting in 3.5 kcal/mol, thus suggesting that the formation of this assembly is dominated by the π-stacking interaction.

**Figure 12:**
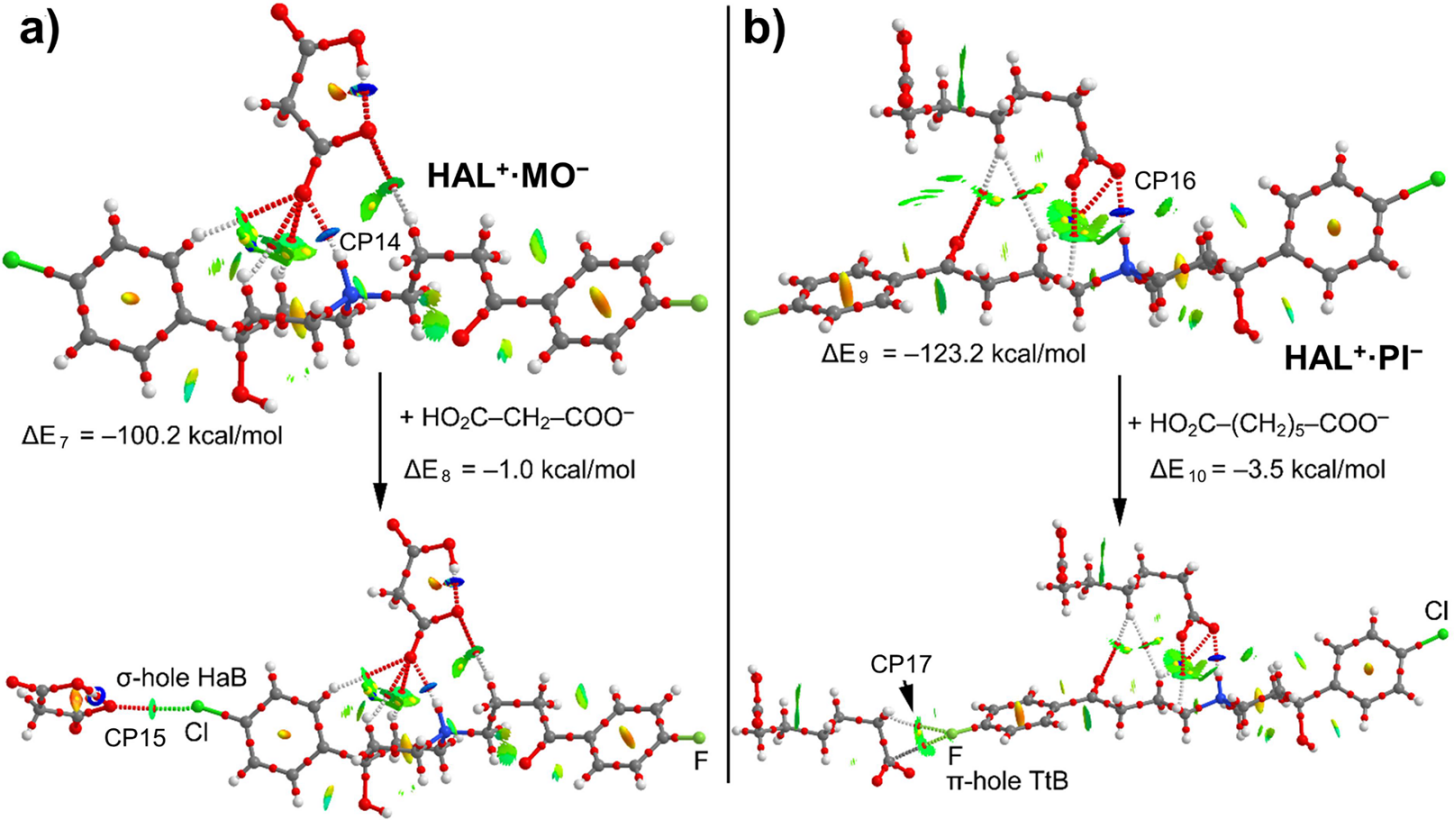
(a) Combined QTAIM (bond and ring CPs in red and yellow, respectively and bond paths) and NCIplot analyses of the salt of compound **HAL**^**+**^**·MO**^**–**^ and the interaction of malonate with the salt. (b) Combined QTAIM (bond and ring CPs in red and yellow, respectively and bond paths) and NCIplot analyses of the salt of compound **HAL**^**+**^**·PI**^**–**^ and the interaction of pimelate with the salt.

Figure 12 shows the QTAIM/NCIplot distribution analyses of compounds **HAL**^**+**^**·MO**^**–**^ and **HAL**^**+**^**·PI**^**–**^, both exhibiting the electrostatically enhanced N^+^–H···^–^OOC H-bond. In fact, the formation energies of both salts are very large (Δ*E*_7_ = −100.2 kcal/mol and Δ*E*_9_ = −123.2 kcal/mol for **HAL**^**+**^**·MO**^**–**^ and **HAL**^**+**^**·PI**^**–**^, respectively) in line with compounds **HAL**^**+**^**·GU**^**–**^ and **HAL**^**+**^**·AD**^**–**^. Both electrostatically enhanced H-bonds are characterized by a bond CP, a bond path connecting the H atom to the O atom and a dark blue isosurface with dissociation energies of 10.5 and 15.3 kcal/mol for **HAL**^**+**^**·MO**^**–**^ and **HAL**^**+**^**·PI**^**–**^, respectively (CP14 and CP15, see Table 4) confirming their strong nature. Interestingly, compound **HAL**^**+**^**·MO**^**–**^, establishes in the solid state a halogen bonding interaction similar to that described above for compound **HAL**^**+**^**·GU**^**–**^. It is characterized by a bond CP and bond path that connect the Cl to the O atom of malonate and a green isosurface indicating a weak interaction. The interaction energy of this halogen bond is Δ*E*_8_ = −1.0 kcal/mol computed by using the reaction shown in Figure 10a. The dissociation energy of this HaB computed using the QTAIM approach (*V*_r_ predictor^73^) is 1.81 kcal/mol, in reasonable agreement to that computed using the supramolecular approach (Δ*E*_8_), thus giving reliability to the *V*_r_ energy predictor (see CP10 in Table 4). Compound **HAL**^**+**^**·PI**^**–**^ (see Figure 12b) forms a π-hole interaction similar to that described for compounds **HAL**^**+**^**·GU**^**–**^ and **HAL**^**+**^**·AD**^**–**^ (see Figure 10). The F atom points to the carbon atom of the carboxylate group of the adjacent molecule. The interaction energy of this motif is Δ*E*_10_ = −3.5 kcal/mol where in addition to the π-hole interaction, an ancillary C–H···O contact is also established as revealed by the combined QTAIM/NCIPlot analysis (CP17). The interaction associated with this contact is small (1 kcal/mol, see Table 4), thus revealing that the π-hole interaction is approximately −2.5 kcal/mol.

Table 4 also gathers the total energy densities (*H*_r_ = *G*_r_ + *V*_r_) measured at the bond CPs labeled in Figures 3–5. They correspond to the H-bonding and HAL-bonding interactions. The *H*_r_ values provide valuable information related to the covalent/noncovalent nature of the interaction. In case the Lagrangian kinetic energy (*G*_r_) is larger than *V*_r_ in absolute value (positive values of *H*_r_,), the interaction is noncovalent. Negative and small values of *H*_r_ (|*V*_r_| > *G*_r_) are indicative of a certain degree of covalency. The *H*_r_ values of Table 4 are negative for the electrostatically enhanced N^+^–H···^–^OOC H-bonds, thus suggesting that their strong nature is due to their partial covalent character in addition to the electrostatic effects.

**Figure 13:**
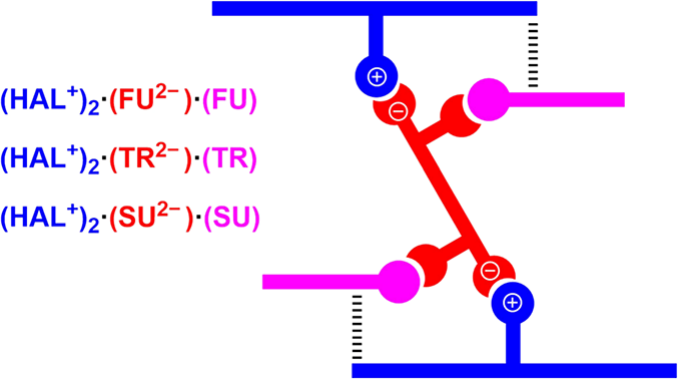
Schematic representation of the hexameric assemblies observed in **(HAL**^**+**^**)**_**2**_**·(FU**^**2–**^**)·(FU)**, **(HAL**^**+**^**)**_**2**_**·(TR**^**2–**^**)·(TR)** and **(HAL**^**+**^**)**_**2**_**·(SU**^**2–**^**)·(SU)** compounds.

For the ionic cocrystals **(HAL**^**+**^**)**_**2**_**·(FU**^**2–**^**)·(FU)**, **(HAL**^**+**^**)**_**2**_**·(TR**^**2–**^**)·(TR)** and **(HAL**^**+**^**)**_**2**_**·(SU**^**2–**^**)·(SU)**, we have focused the analysis on the differences between the strong N–H···O and O–H···O H-bonds and the formation energies of the hexameric assemblies schematically represented in Figure 13, which are common in the solid state of the three compounds.

**Figure 14:**
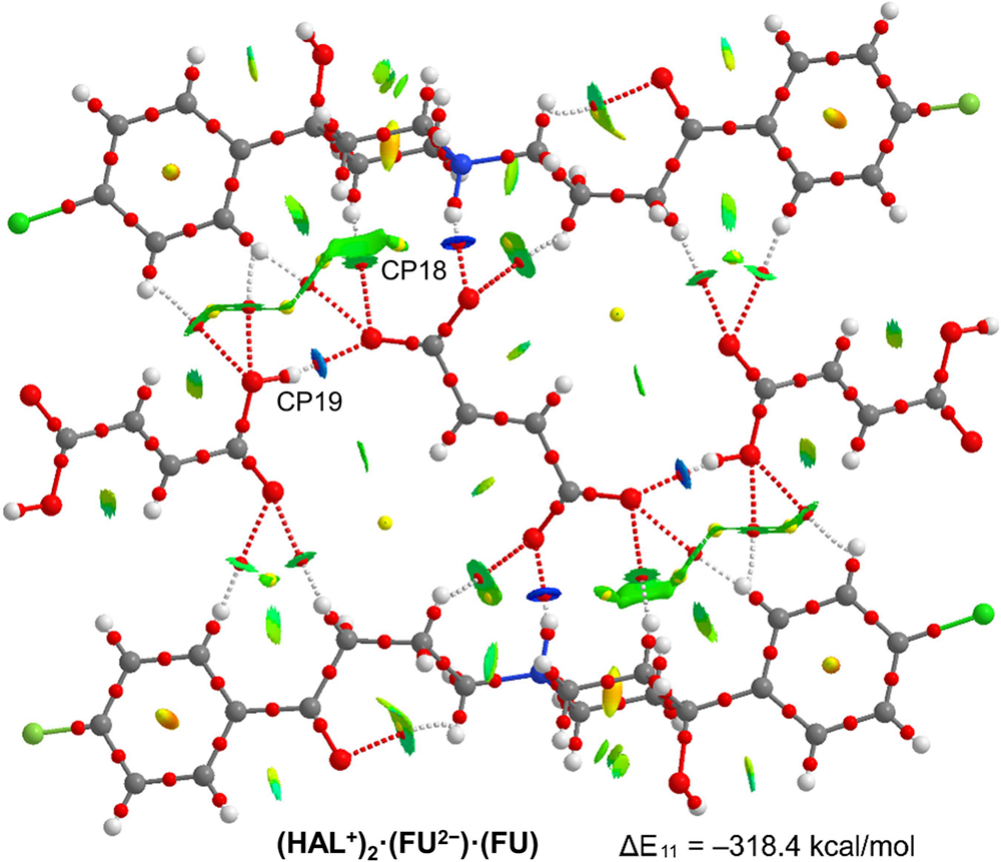
Combined QTAIM (bond and ring CPs in red and yellow, respectively and bond paths) and NCIplot analyses of the salt of compound **(HAL**^**+**^**)**_**2**_**·(FU**^**2–**^**)·(FU)** and the formation energy of the assembly.

Figure 14 shows the assembly of **(HAL**^**+**^**)**_**2**_**·(FU**^**2–**^**)·(FU)** where the fumarate dianion connects two **HAL**^**+**^ units by N–H···O and C–H···O H-bonds. Moreover, the dianion also interacts with two neutral fumaric acids. The formation energy of the assembly is very large (Δ*E*_11_ = −308.4 kcal/mol) due to the electrostatic component and the contribution of the four strong H-bonds. These H-bonds are characterized by the corresponding bond CPs, bond paths and blue isosurfaces (see CP18 and CP19 in Figure 14) connecting the H and O-atoms. The energy associated to these H-bonds (see Table 4) are 13.9 and 10.9 kcal/mol for N–H···O and O–H···O respectively. The combined QTAIM/NCIplot analysis shows the existence of a network of C–H···O H-bonds that further contribute to the stabilization of the assembly.

**Figure 15:**
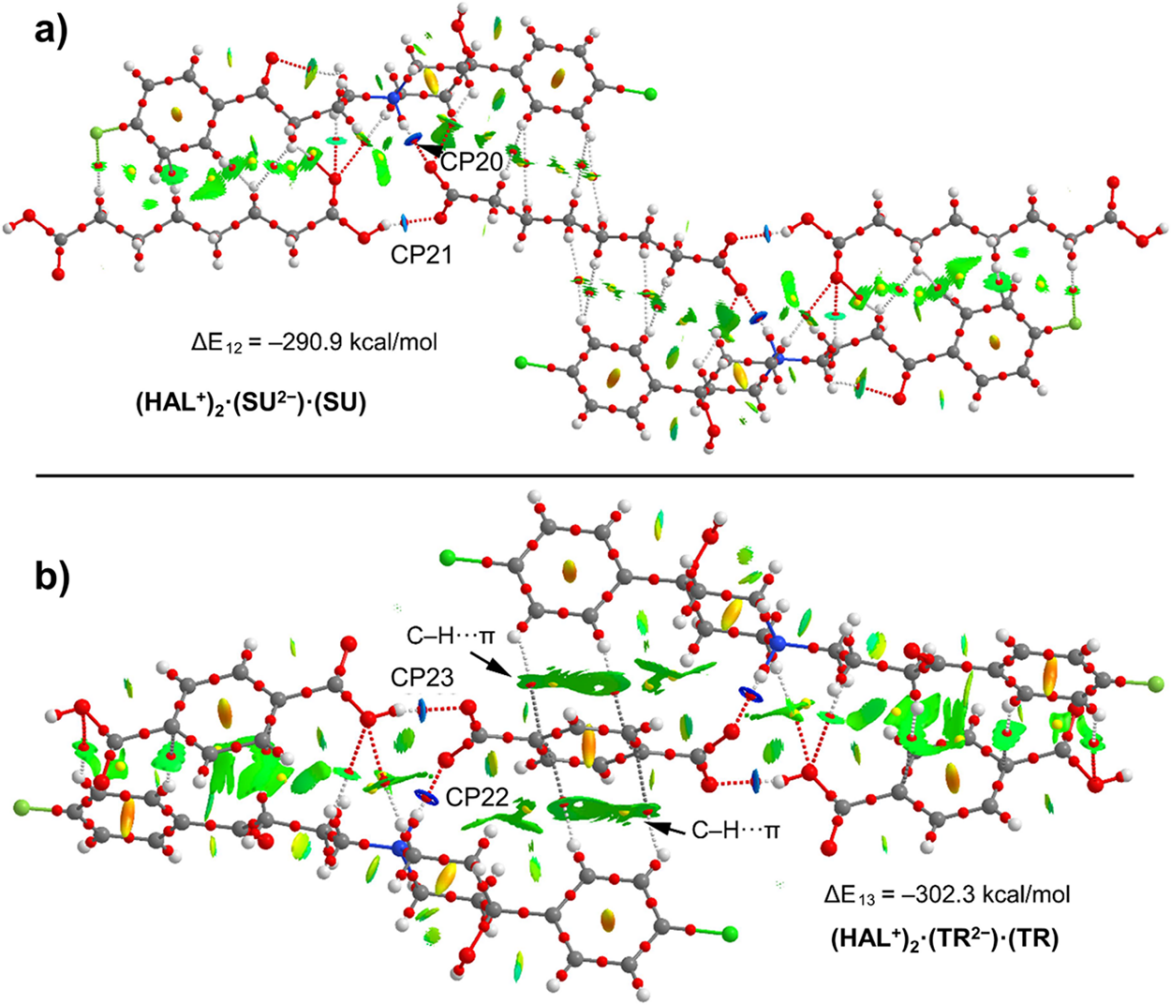
Combined QTAIM (bond and ring CPs in red and yellow, respectively and bond paths) and NCIplot analyses of compounds **(HAL**^**+**^**)**_**2**_**·(SU**^**2–**^**)·(SU)** (top) and **(HAL**^**+**^**)**_**2**_**·(TR**^**2–**^**)·(TR)** (bottom) and their respective formation energies.

Finally, Figure 15 shows the assemblies of **(HAL**^**+**^**)**_**2**_**·(SU**^**2–**^**)·(SU)** and **(HAL**^**+**^**)**_**2**_**·(TR**^**2–**^**)·(TR)** where the subarate and terephtalate dianions, respectively, interconnect both HAL^+^ units by strong N^+^–H···O H-bonds. The calculated formation energies of the hexameric assemblies are Δ*E*_12_ = −290.9 kcal/mol and Δ*E*_13_ = −302.3 kcal/mol for **(HAL**^**+**^**)**_**2**_**·(SU**^**2–**^**)·(SU)** and **(HAL**^**+**^**)**_**2**_**·(TR**^**2–**^**)·(TR)**, respectively. These are smaller in absolute value than the formation energy of the hexameric assembly of **(HAL**^**+**^**)**_**2**_**·(FU**^**2–**^**)·(FU)**, because the much-reduced network of C–H···O H-bonds. The QTAIM/CNI plot analyses show the existence of other weaker interactions like C–H···H–C in **(HAL**^**+**^**)**_**2**_**·(SU**^**2–**^**)·(SU)** involving the methylene groups of SU or C–H···π in **(HAL**^**+**^**)**_**2**_**·(TR**^**2–**^**)·(TR)** involving the π-system of TR. The energy associated to the N^+^–H···O H-bonds (see Table 4) are 11.5 and 15.7 kcal/mol for **(HAL**^**+**^**)**_**2**_**·(SU**^**2–**^**)·(SU)** and **(HAL**^**+**^**)**_**2**_**·(TR**^**2–**^**)·(TR)** respectively and those for the O–H···O H-bonds are slightly weaker (10.1 and 10.7 kcal/mol, respectively). The QTAIM values of *H*_r_ (see Table 4) are positive for the O–H···O H-bonds and negative for the N^+^–H···O ones, thus confirming the partial covalent character of the latter. When compared to conventional salts, the highly stable arrangement observed in ionic cocrystals is expected to have a significant impact on the following physicochemical properties characterization, particularly in terms of thermal and thermodynamic stability, as well as solubility.

### 3.5 Thermal Stability

**Figure 16:**
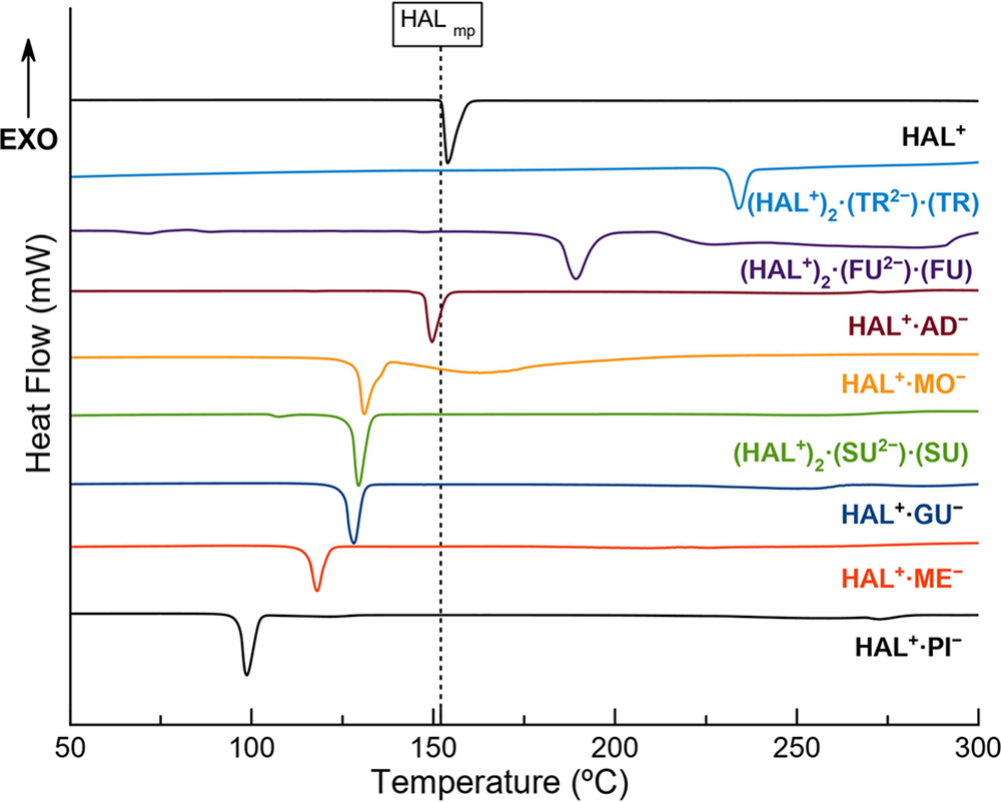
DSC traces of HAL and the new solids obtained in this work. Black-dotted line correspond to the melting point of HAL.

The thermal stability of HAL and the reported pharmaceutical salts and ionic cocrystals was evaluated by DSC, as shown in Figure 16. The DSC traces reveal a single and well-defined endothermic peak for each compound, indicating the purity of the bulk product obtained by LAG (also corroborated by PXRD) and the thermal stability of the novel materials. Additionally, no transitions between crystalline phases are observed before melting, except for **(HAL^+^)_2_·(SU^2–^)·(SU)**, which shows a small endothermic signal that may be attributed to a phase transition, and **(HAL^+^)_2_·(FU^2–^)·(FU)**, where the signals are attributed to moisture loss. These results are supported by TGA analysis, no mass loss is observed for **(HAL^+^)_2_·(SU^2–^)·(SU)**, supporting the phase transition hypothesis, while a weight loss of 0.95% is observed for **(HAL^+^)_2_·(FU^2–^)·(FU)**, consistent with moisture loss from the sample. Thermal events associated with the degradation of the samples are also observed beyond the melting points, also corroborated by TGA analysis, in which mass loss only occurred after the melting point (Figure S2).

These results show no apparent relation between the coformer length and the melting point of the phase (Table 5). For instance, MO (C3) produces a salt with a similar melting point (**HAL**^**+**^**·MO**^**–**^_mp_:131.4 °C) compared to **HAL**^**+**^**·GU**^**–**^ (C5) (128.4 °C), despite having fewer methylene groups. Similarly, SU (C8) forms an ionic cocrystal with a lower melting point (**(HAL**^**+**^**)**_**2**_**·(SU**^**2–**^**)·(SU)**_mp_:129.4 °C) than **HAL**^**+**^**·AD**^**–**^ (149.6 °C). On the contrary, a clear relationship between the melting point of the coformer molecule and the melting point of its corresponding phase is observed. This is evident in the cases of FU and TR, whose ionic cocrystals display the highest melting points (**(HAL**^**+**^**)**_**2**_**·(FU**^**2–**^**)·(FU)**_mp_:189.7 °C; **(HAL**^**+**^**)**_**2**_**·(TR**^**2–**^**)·(TR)**_mp_:234.7 °C), aligning with the melting point of their coformers. Conversely, **(HAL**^**+**^**)**_**2**_**·(SU**^**2–**^**)·(SU)** exhibits a significantly lower melting point (129.4 °C), consistent with the lower melting point of SU. These results indicate that it is not necessarily the higher lattice energy of ionic cocrystals what determines a higher melting point, but rather the thermal properties of its coformers. Although the salt involving glutaric acid deviates from this trend, the behavior observed in all the novel materials aligns with other reported studies in the area.^68,74,75^

**Table 5:** Melting Points of Coformer Molecules and Their Respective PMMs, Arranged by Coformer Melting Point

coformer	melting point (°C)	salt	melting point (°C)
Pimelic Acid (PI)	103–105	**HAL**^**+**^**·PI**^**–**^	98.7
Maleic Acid (ME)	130.5	**HAL**^**+**^**·ME**^**–**^	118.1
Malonic Acid (MO)	132–135	**HAL**^**+**^**·MO**^**–**^	131.4
Suberic Acid (SU)	140–144	**(HAL**^**+**^**)**_**2**_**·(SU**^**2–**^**)·(SU)**	129.4
Adipic Acid (AD)	152.1	**HAL**^**+**^**·AD**^**–**^	149.6
Glutaric Acid (GU)	200	**HAL**^**+**^**·GU**^**–**^	128.4
Fumaric Acid (FU)	287	**(HAL**^**+**^**)**_**2**_**·(FU**^**2–**^**)·(FU)**	189.7
Terephthalic Acid (TR)	427	**(HAL**^**+**^**)**_**2**_**·(TR**^**2–**^**)·(TR)**	234.7

### 3.6 Thermodynamic Stability

The thermodynamic stability of the salts was assessed in aqueous media under accelerated aging conditions (40 °C, 75% RH, Figure S3) and at physiologically relevant pH values (PBS pH 6.8 and KCl pH 1.2, Figure S4) to discard processes such as dissociation, hydration, or polymorphic transitions.

In these studies, the stability of the salts was evidenced, as no significant changes in the diffraction patterns or crystallinity were observed, except for the **HAL**^**+**^**·ME**^**–**^, which underwent a transition to another crystalline form under all conditions. In fact, under accelerated aging conditions, the phase transition occurred during the first 24 h of exposition. This instability can be attributed to the reported potential for multiple polymorphic forms ME-salts that may lead to phase transitions under stress conditions.^76−78^ Moreover, the inherent hygroscopic nature of maleic acid and being the strongest acid among the coformers (p*K*_a1_ = 1.94 and p*K*_a2_ = 6.22) can lead to pharmaceutical salts that may exhibit increased sensitivity to humidity and low pH environments.^79,80^ This phenomenon has been observed in other pharmaceutical compounds where maleic acid salts showed more degradation during stability studies, particularly under acidic conditions.^81^

### 3.7 Solubility

The solubility of HAL obtained with the novel solids was assessed by HPLC in aqueous media at two pH conditions: acidic (KCl, pH 1.2) and near-neutral (PBS, pH 6.8), to simulate the conditions found in the gastrointestinal tract. These results are presented in Table 6, highlighting the impact of salt formation on the aqueous solubility of HAL. Despite the observed crystalline phase transition in **HAL**^**+**^**·ME**^**–**^, no evidence of degradation was detected, as neither HAL, coformer ME, or any known polymorphic or hydrate form of the components were observed via PXRD. This suggests that the transition is likely due to hydration or polymorphic transformation rather than dissociation of the salt. Given these stability considerations, the solubility of **HAL**^**+**^**·ME**^**–**^ was further investigated.

**Table 6:** HPLC Measurements of HAL Concentrations at 248 nm

compound	solubility in KCl pH 1.2 (mg/mL)	improvement over HAL	solubility in PBS pH 6.8 (mg/mL)	improvement over HAL
**HAL**	0.831 ± 0.042		0.007 ± 0.001	
**HAL**^**+**^**·MO**^**–**^	0.525 ± 0.037	0.6×	0.119 ± 0.002	15.9×
**HAL**^**+**^**·ME**^**–**^	1.401 ± 0.020	1.7×	0.074 ± 0.004	9.9×
**(HAL**^**+**^**)**_**2**_**·(FU**^**2–**^**)·(FU)**	1.056 ± 0.027	1.3×	0.033 ± 0.004	4.5×
**HAL**^**+**^**·GU**^**–**^	1.021 ± 0.012	1.2×	0.048 ± 0.001	6.4×
**HAL**^**+**^**·AD**^**–**^	0.285 ± 0.079	0.3×	0.012 ± 0.001	1.6×
**(HAL**^**+**^**)**_**2**_**·(TR**^**2–**^**)·(TR)**	0.678 ± 0.019	0.8×	0.051 ± 0.002	6.8×
**HAL**^**+**^**·PI**^**–**^	1.246 ± 0.035	1.5×	0.072 ± 0.001	9.7×
**(HAL**^**+**^**)**_**2**_**·(SU**^**2–**^**)·(SU)**	1.074 ± 0.036	1.3×	0.017 ± 0.001	2.3×

Under acidic conditions (KCl pH 1.2) no clear relationship between the coformer (its length or acidity) and the solubility of the novel material was observed. Under these conditions, the novel materials allowed to increase the solubility of HAL (0.831 mg/mL) up to 1.401 and 1.246 mg/mL in the case of **HAL**^**+**^**·ME**^**–**^ and **HAL**^**+**^**·PI**^**–**^ respectively. A moderate improvement of solubility is also observed with **(HAL**^**+**^**)**_**2**_**·(FU**^**2–**^**)·(FU)**, **HAL**^**+**^**·GU**^**–**^ and **(HAL**^**+**^**)**_**2**_**·(SU**^**2–**^**)·(SU)**, which present solubilities close to 1 mg/mL. On the other hand, **(HAL**^**+**^**)**_**2**_**·(TR**^**2–**^**)·(TR), HAL**^**+**^**·MO**^**–**^ and **HAL**^**+**^**·AD**^**–**^ decrease the solubility of HAL down to 0.678, 0.525, and 0.285 respectively.

Despite the greater absolute increase in solubility at acidic pH with certain salts, the true percentage improvement compared to HAL is observed under the neutral conditions, present in the intestinal tract, in which HAL presents the major drawbacks of solubility. In these conditions, our results show that all salts exhibited significantly improved solubility compared to pure HAL, which is almost insoluble (maximum concentration of 0.007 mg/mL at physiological conditions), in line with previously reported results.^5,6,41^ Interestingly, such improvement was most notable with short-length coformers, MO (C2) and ME (C3), whose salts exhibited solubility enhancements of 15.9×, 9.9×, respectively. This issue is a consequence of the lower lattice enthalpy observed in the DFT studies, and the separation of the molecules of HAL^+^, which facilitates crystal disruption. These improvements are expected to enhance the oral bioavailability and intestinal absorption of HAL when administered orally.

In contrast, TR and FU form ionic cocrystals with high stability due to π···π stacking and robust hydrogen bonding networks, leading to significantly lower solubility. This is especially notable when comparing the solubility of **(HAL**^**+**^**)**_**2**_**·(FU**^**2–**^**)·(FU)** with **HAL**^**+**^**·ME**^**–**^, both phases with C4 coformers, with the solubility of the salt being twice the solubility of the ionic cocrystal. However, this trend is not observed in the case of the phases with C6 coformers. Comparing the solubility of **HAL**^**+**^**·AD**^**–**^ to **(HAL**^**+**^**)**_**2**_**·(TR**^**2–**^**)·(TR)**, the salt is 4 times lower than the ionic cocrystal.

Although no definitive explanation has been found to explain the unexpectedly low solubility of **HAL**^**+**^**·AD**^**–**^ (also observed under acidic conditions), the overall results show higher solubility, in terms of percentage of improvement, under PBS pH 6.8. This improvement is enhanced when the salts are formed by short-length coformers, while for medium-to-long-chain coformers, salt formation generally leads to a more significant solubility improvement compared to the corresponding ionic cocrystals. These results align with expectations based on SCXRD and DFT analyses, which suggest that the structural and energetic properties of the salts facilitate improved dissolution behavior.

## 4 Conclusions

In this study five pharmaceutical salts and three ionic cocrystals of HAL with variable-length dicarboxylic acids have been synthesized by LAG and subsequently confirmed through SCXRD analysis. Additional DFT calculations and QTAIM/NCIplot analyses of the noncovalent interaction were used to assess the influence of coformer length on the final properties of the material.

Unexpectedly, our findings indicate that maximizing the solubility of HAL under neutral conditions, critical for improving intestinal absorption in oral administration, is best achieved by selecting small, highly reactive molecules that yield pharmaceutical salts. This approach enhances the disruption of HAL native structure, leading to increased solubility. Interestingly, the use of longer-chain coformers does not necessarily result in greater molecular separation. Instead, due to their size, they tend to align in parallel chains with HAL forming more cohesive structures stabilized by additional noncovalent interactions, leading to materials with higher thermal stability but lower aqueous solubility.

Additionally, understanding the factors that determine whether a system forms a salt or an ionic cocrystal is crucial, particularly when the Δp*K*_a_**rule** fails. Deviations from this rule were observed, in the cases of **(HAL**^**+**^**)**_**2**_**·(FU**^**2–**^**)·(FU), (HAL**^**+**^**)**_**2**_**·(TR**^**2–**^**)·(TR)**, and **(HAL**^**+**^**)**_**2**_**·(SU**^**2–**^**)·(SU)**, where ionic cocrystals were formed despite Δp*K*_a_ > 4, which typically favors salt formation. In these systems, double deprotonation of the dicarboxylic acid plays a key role, influenced by factors such as coformer geometry, resonance stabilization, and hydrogen bonding patterns. For instance, the cis-isomer ME forms a salt due to intramolecular hydrogen bonding, which stabilizes its monoanionic form, whereas trans-isomer FU promotes ionic cocrystal formation by facilitating double deprotonation. Similarly, TR benefits from π-electron delocalization, which enhances deprotonation and leads to ionic cocrystal formation.

Given the significance of our findings, further investigations should explore additional coformer families, including aromatic compounds with resonance features (i.e., polyphenols, dihydroxybenzoic acids), coformers with different functional groups (i.e., amino acids, basic molecules), and compounds with diverse geometries and electronic effects. Studying these alternative coformers will provide deeper insight into coformer-API interactions, ultimately optimizing the physicochemical properties of pharmaceutical multicomponent materials for specific applications.

## Supporting Information Available

C–O bond distances of HAL multicomponent materials; Fourier difference map of the reported compounds showing an electron density peak between the O···N atoms; DSC and TGA traces of HAL multicomponent materials; and PXRD patterns showing the stability in PBS (pH 6.8) and KCl (pH 1.2) (PDF)
