# Supplementary material for: Impact of the Coformer Carbon-Chain Length on the Properties of Haloperidol Pharmaceutical Salts
Source: Cryst Growth Des. 2025 Apr 28;25(9):3169–85. doi: 10.1021/acs.cgd.5c00251 (PMC12063055; doi:10.1021/acs.cgd.5c00251)
Supplement: Supplementary file 1 — cg5c00251_si_001.pdf [file cg5c00251_si_001.pdf]

Supporting Information

Impact of the coformer carbon-chain length on the properties of Haloperidol pharmaceutical salts.

Francisco J. Acebedo-Martínez,[1] *Carolina Alarcón-Payer*[2], *Alicia Domínguez-Martín*[3], *Antonio Frontera*[4], *Cristóbal Verdugo-Escamilla*[1], *Duane Choquesillo-Lazarte*[1,*]

[1] Laboratorio de Estudios Cristalográficos, IACT-CSIC, Avda. de las Palmeras 4, 18100 Armilla, Spain.

[2] Servicio de Farmacia, Hospital Universitario Virgen de las Nieves, 18014 Granada, Spain.

[3] Department of Inorganic Chemistry, Faculty of Pharmacy, University of Granada, 18071 Granada, Spain.

[4] Departament de Química, Universitat de les Illes Balears, Crta. de Valldemossa km 7.5, 07122 Palma, Spain.

Table S1. C-O bond distance of HAL multicomponent materials.

Compound		Δ_{C-O}
**$HAL^{+}\cdot MO^{-}$**		
O(3)-C(22)	1.225(3)	0.028
O(4)-C(22)	1.253(3)	
**$HAL^{+}\cdot ME^{-}$**		
O(3)-C(22)	1.244(3)	0.019
O(4)-C(22)	1.263(4)	
**$(HAL^{+})_2\cdot(FU^{2-})\cdot(FU)$**		
C(24)-O(5)	1.257(4)	0.006
C(24)-O(6)	1.263(4)	
**$HAL^{+}\cdot GU^{-}$**		
C(22)-O(3)	1.266(7)	0.049
C(22)-O(4)	1.217(7)	
**$HAL^{+}\cdot AD^{-}$**		
O(5)-C(27)	1.237(4)	0.027
O(6)-C(27)	1.264(4)	
**$(HAL^{+})_2\cdot(TR^{2-})\cdot(TR)$**		
O(3)-C(22)	1.250(3)	0.020
O(4)-C(22)	1.270(3)	
**$HAL^{+}\cdot PI^{-}$**		
O(3)-C(22)	1.268(8)	0.030
O(4)-C(22)	1.238(8)	
**$(HAL^{+})_2\cdot(SU^{2-})\cdot(SU)$**		
O(5)-C(26)	1.254(2)	0.005
O(6)-C(26)	1.249(2)	

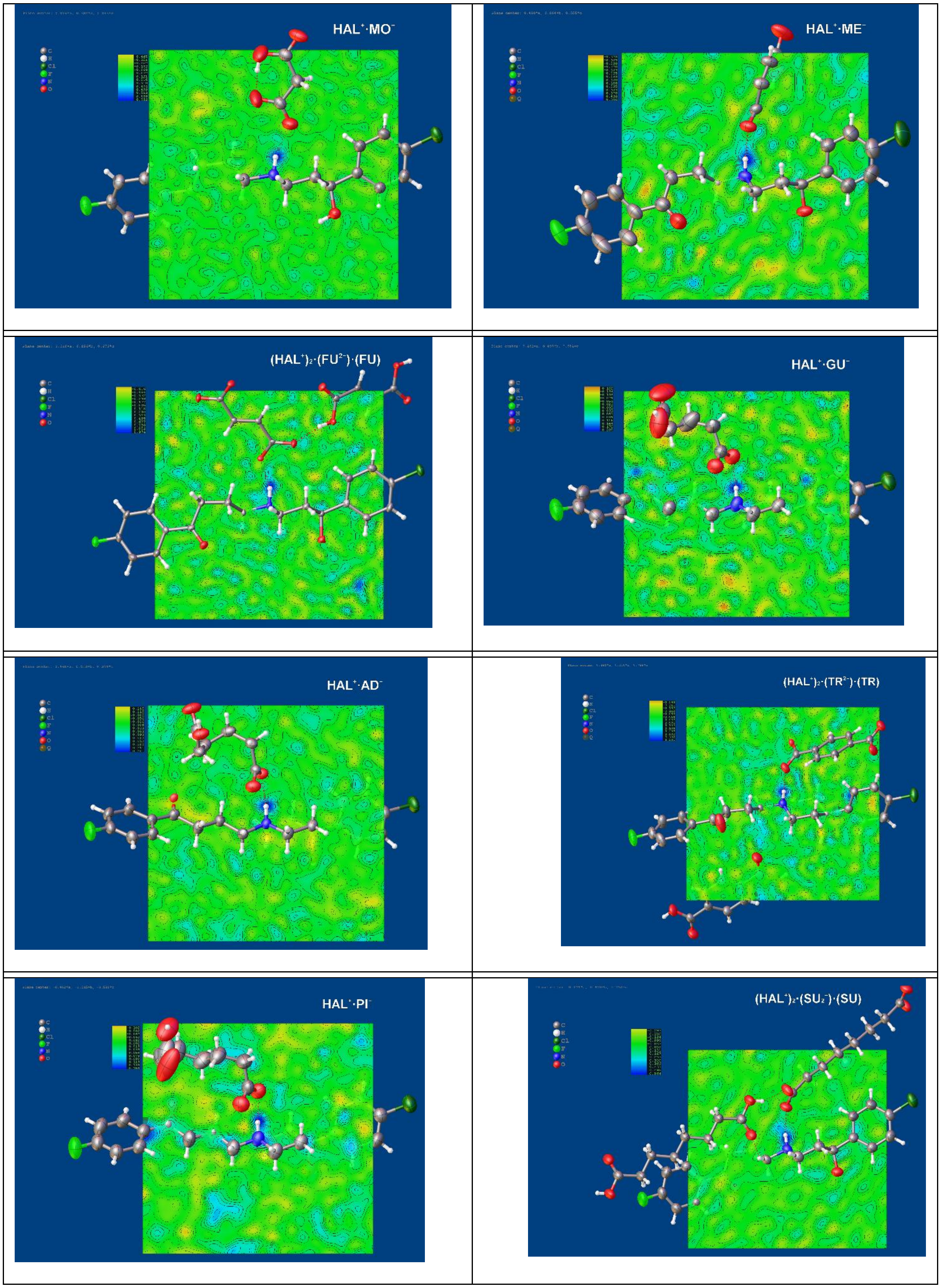

Figure S1. Fourier difference map of the reported compounds showing an electron density peak (blue) between the O···N atoms due to proton transfer from carboxylic groups to amines in salt formation.

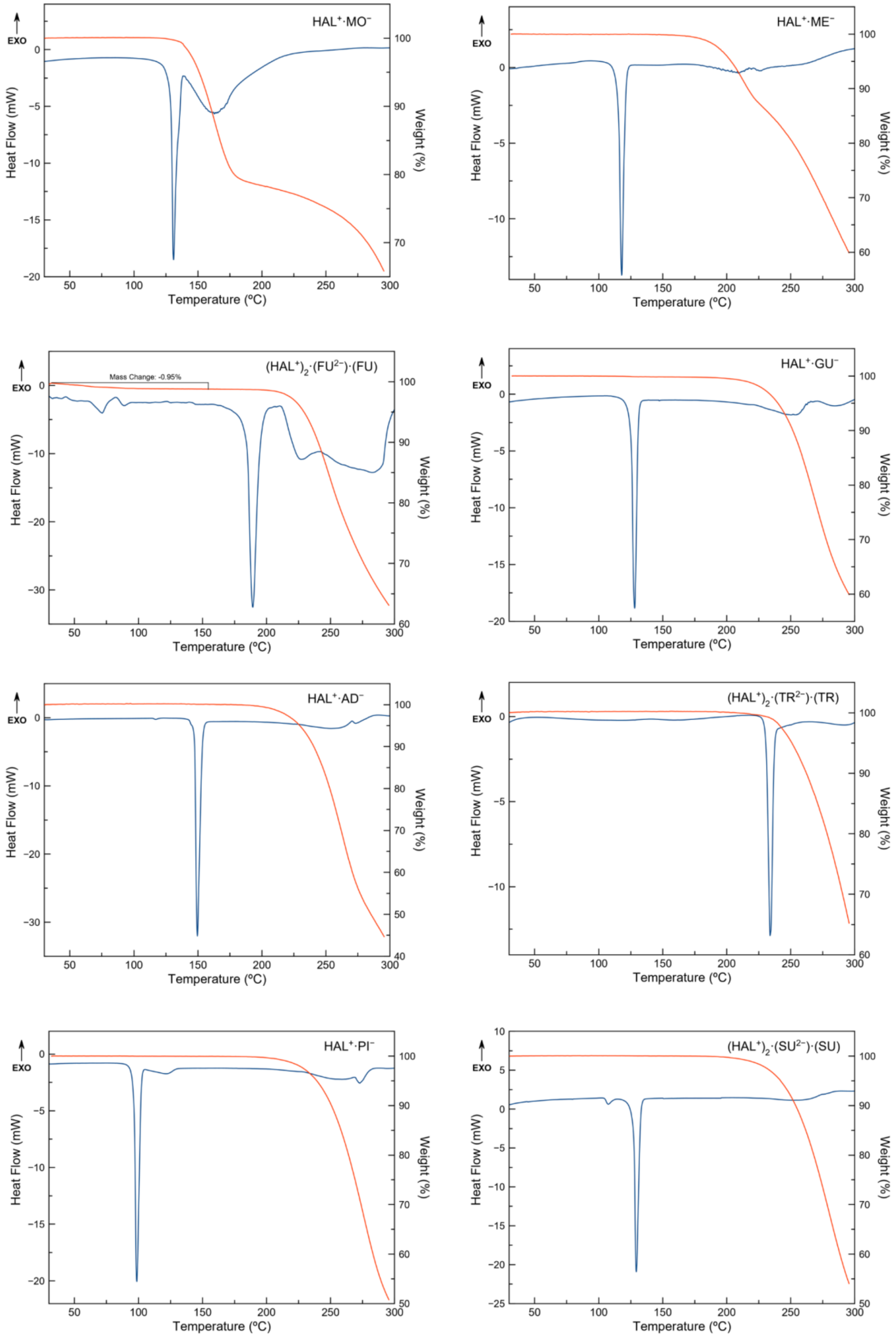

Figure S2. DSC and TGA traces of HAL multicomponent materials.

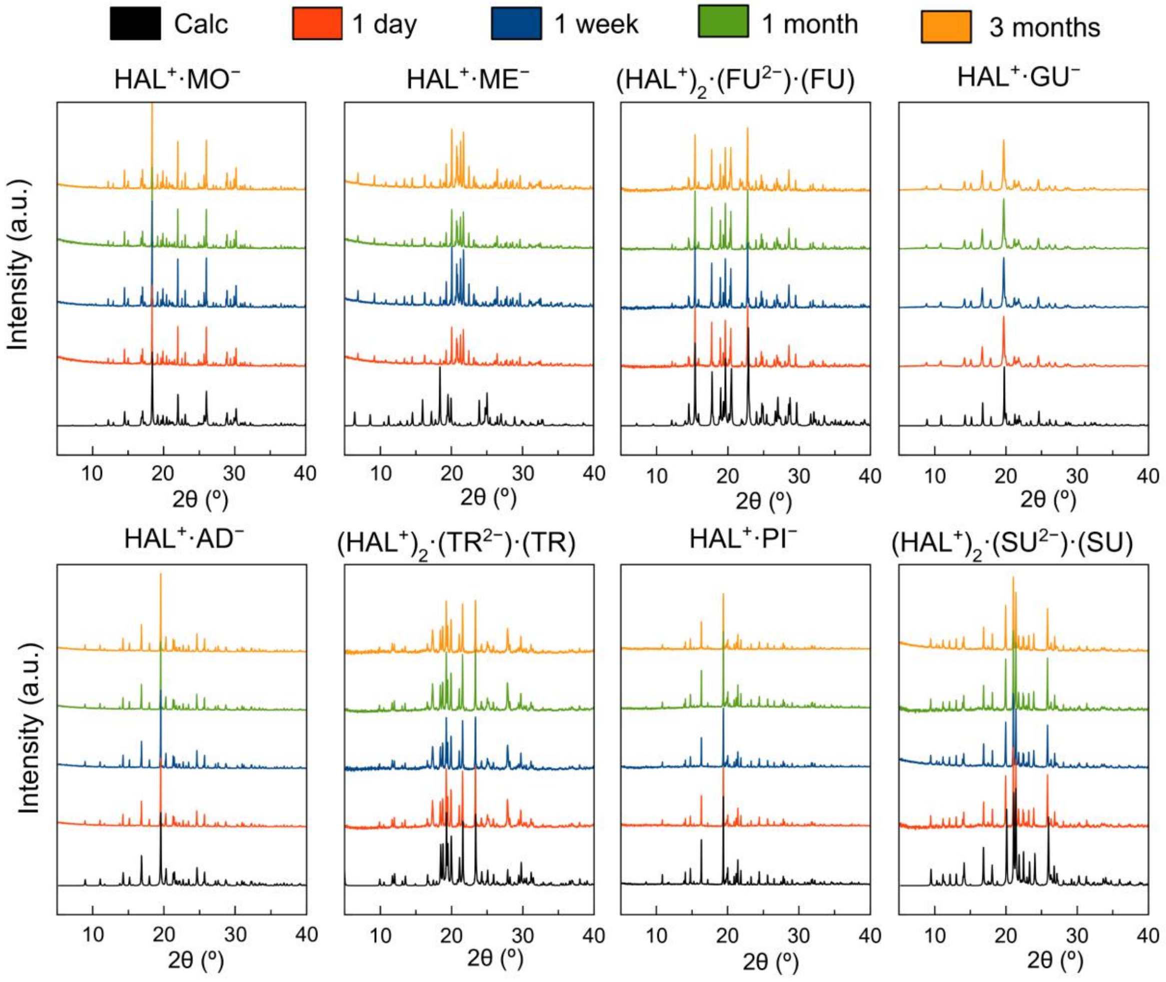

Figure S3. PXRD patterns of the novel phases showing the stability under accelerated ageing conditions (40 ºC, 75 % RH)

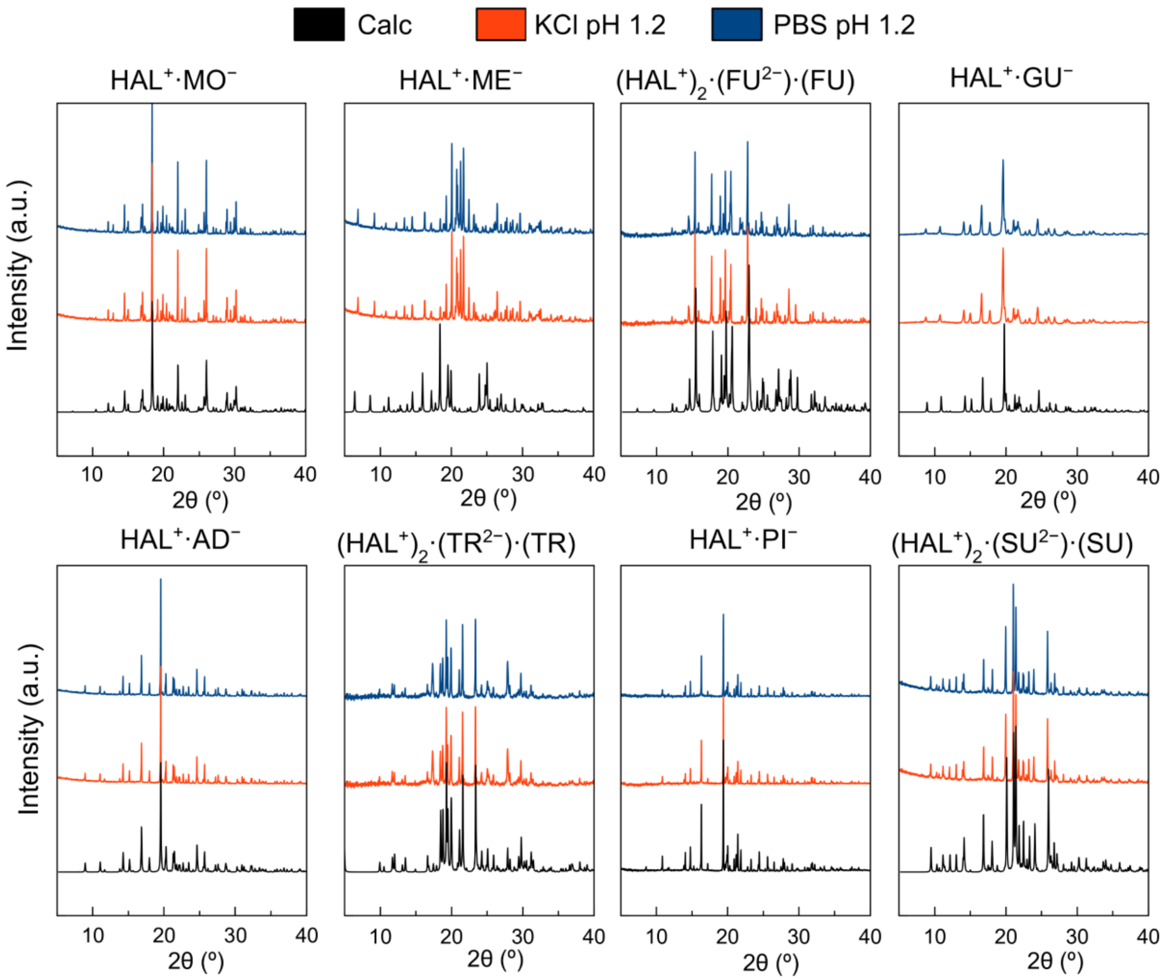

Figure S4. PXRD patterns of the novel phases showing the stability in PBS (pH 6.8) and KCl (pH 1.2)
